# A data-driven methodological framework for representative recruitment in psychiatric research: insights from the DOCUMENT study

**DOI:** 10.1186/s12888-026-08442-x

**Published:** 2026-07-31

**Authors:** Benjamin J. M. Gooddy, Laila Rida, Bryony Goulding Mew, Ana Rita Moura, Timea Szentgyorgyi, Brandi Eiff, Luisa Schalk, Iman Rafiq, Cathy Davies, Daniel Martins, Lilla A. Porffy, Christabel Gibson, Caroline Wooldridge, Giorgio Bergamini, Janet R. Nicholson, Markus Waser, Elizabeth Tunbridge, Sigurd D. Süssmuth, Valdemar Robert Janulczyk, Karla V. Allebrandt, Adam Hampshire, Sukhwinder S. Shergill, Steve Williams

**Affiliations:** 1 Centre for Neuroimaging Sciences, King’s College London - Institute of Psychiatry, Psychology & Neuroscience, London, UK; 2 Department of Psychosis Studies, Institute of Psychiatry, Psychology & Neuroscience, King’s College London, London, UK; 3 Boehringer Ingelheim Pharma GmbH & Co. KG, Biberach an der Riss, Germany; 4 Kent and Medway Medical School, Canterbury, UK; 5 Kent and Medway National Health Service and Social Care Partnership Trust, Kent, UK

**Keywords:** Participant recruitment, Depression, Psychosis, Schizophrenia, Sampling bias, Psychiatric research, Major depressive disorder, Clinical study, Digital health, Clinical research

## Abstract

**Background:**

Slow participant recruitment is one of the predominant determinants of failure or delay across clinical research. Even when recruitment targets are met, study populations may be unrepresentative due to sampling biases introduced by recruitment pathways. However, the effectiveness and demographic consequences of recruitment strategies are frequently underreported, undermining the generalisability of clinical findings and contributing to research waste. This study provides a data-driven, quantitative evaluation of multimodal recruitment strategies in psychiatric research, leveraging insights from the DOCUMENT study to synthesise a methodological framework for effective and representative participant recruitment.

**Methods:**

Between June 2022 and December 2024, the study utilised a multimodal strategy to recruit participants with major depressive disorder (MDD), schizophrenia (SZ), and healthy volunteers (HV) for a two-phase study to investigate cognitive deficits across groups. Recruitment strategies included NHS clinical services, electronic health records, research registries, primary care sites, online and social media advertising, printed material, institutional resources, and word of mouth. For each avenue, yield, proportion of diagnostic group, recruitment rate, eligibility fraction, labour and financial cost, and demographic skew were quantified.

**Results:**

Across avenues, 194 participants were recruited (66 HV, 77 MDD and 51 SZ), with high retention (86–97%). Recruitment efficacy varied substantially by diagnostic group, with online advertising and research registries successfully recruiting MDD and HV participants but failing to recruit eligible people with SZ. Instead, SZ participants were primarily enrolled from labour-intensive clinical avenues (94%). Online recruitment showed higher accrual, but lower eligibility fraction compared to clinical pathways, revealing systematic sampling differences. Despite avenue-specific sampling biases, the multimodal approach yielded close demographic alignment to the 2021 UK Census for London in the study population. Data-driven adaptations, such as protocol amendments to eligibility criteria and online self-report triaging, improved study feasibility.

**Conclusions:**

No single recruitment avenue was identified as sufficient for both efficient and representative psychiatric recruitment. Instead, multimodal strategies were necessary to dilute avenue sampling biases. Synthesising 30 months of data, we introduce a 10-point framework for enhancing recruitment effectiveness, feasibility, and representativeness. While grounded in UK-based psychiatric research, these principles are likely transferable to broader clinical research contexts to reduce research waste.

**Clinical trial number:**

Not applicable.

**Supplementary Information:**

The online version contains supplementary material available at https://doi.org/10.1186/s12888-026-08442-x.

## Background

Participant recruitment for clinical research is notoriously difficult, with recruitment issues accounting for ~ 54% of all study extensions, serving as the largest reason for protocol amendments and study discontinuation of randomised clinical trials in particular [2, 24]. Estimates of studies funded in the UK by bodies such as the Health Technology Assessment (HTA) and Medical Research Council (MRC) indicate that only around half (31–56%) of studies reach their full recruitment target and only 55–79% achieve > 80% [27, 42]. This has the serious, wider knock-on effect of potentially underpowered trials with increased false error rates [26].

Recruitment issues can have serious implications, both for study quality and financial feasibility. For example, a failure to meet recruitment targets may induce unintended consequences that undermine the quality of a trial’s data. Attempts to remedy recruitment shortfalls by quickly onboarding additional recruitment sites, which often contribute small numbers of patients, risk increasing the variability in both study baseline and outcome measures across sites [11, 32]. This sampling-induced heterogeneity may increase endpoint variability, complicating the interpretation of trial findings, and ultimately increasing the risk of study failure.

Recruitment challenges can have substantial financial implications: economic modelling of proxy placebo-controlled surgical trials indicates additional costs of up to 50% for trials requiring extension through additional time or sites, and up to 260% above budget for incomplete trials [38]. For example, recruitment issues relating to study discontinuation were costing one medical and academic research site in Oregon an estimated $1 million US dollars a year [22].

Recruitment is a pervasive challenge across clinical fields, from oncology and obstetrics to neurology and psychiatry [12, 34]. However, despite the difference in clinical focus, the same core reasons underlie recruitment failure, including excessively stringent and unrealistic recruitment criteria, overly optimistic recruitment rate projections, insufficient or mishandled budgets, participant burden disproportionate to remuneration, inadequate manpower, trial fatigue, and ineffective recruitment strategy [2, 26]. However, with informed insights, thoughtful strategy, and realistic study design planning based on reflections of previous research experience, many of these issues can be mitigated before a study even begins. Nevertheless, psychiatric research studies face additional unique challenges, such as the reliance on syndromic diagnoses rather than diagnostic biomarkers and less structured clinical pathways for patients, which make it a challenge to identify potential participants and exacerbate recruitment complexity. For example, diagnostic instability – such as from non-affective to affective psychosis – can complicate the recruitment of a stable study population [30].

The unique logistical and sampling requirements of recruiting and retaining eligible psychiatric study populations are exemplified by studies aiming to recruit people with major depressive disorder (MDD) or psychosis. Depression studies often face issues recruiting due to factors such as public scepticism and stigma, lack of patients’ self-identification or insight into the disorder, and instability of symptom severity and clinical stages, such as requiring participants to be in a depressive episode or pre-onset [6, 23]. Stringent inclusion criteria may further exacerbate recruitment issues and even instil an unintentional sampling bias into the study. For example, a study trialling the self-enrolment of depressed participants found that around 25% of patients had never been to their GP about their depression – a population that would be missed if a formal diagnosis was required for inclusion ([3]. Studies recruiting psychosis patients – particularly schizophrenia – are subject to even greater difficulties, in part due to the pronounced complex clinical, functional and cognitive burden of the disorder. Barriers to recruitment include a time-consuming and labour-intensive process of finding patients from clinical records, patients’ inability to participate due to illness severity, particularly in cases of high study burden [45], and questions over patients’ capacity, and gatekeeping by mental health and care professionals not wishing to add to patient burden [9, 35].

Even when recruitment targets are met, it is difficult to determine whether the resultant clinical study sample is representative of the underlying clinical population, due to the risk of recruitment sampling bias. Assessing the scope of sampling bias in the study population, by direct comparison to the real-world clinical population, becomes complicated due to study-specific inclusion and exclusion criteria (e.g. comorbid psychiatric conditions, medication use etc.), which intrinsically may limit representativeness. The identification and evaluation of these sampling biases can be obscured in the absence of comprehensive and transparent reporting of study recruitment methodology.

Despite the push for transparent recruitment practices, such as The Consolidated Standards of Reporting Trials (CONSORT) requiring RCTs to report eligibility criteria and participant attrition, current estimates indicate that adequate compliance with these standards only occurs ~ 63% of the time, and in multi-site studies this drops further to ~ 25% [42]. Representativeness of the study sample to the population of the specific diagnostic group in the real world is fundamental for the extrapolation and generalisation of the research findings [41, 46].

The inherent complexity in reaching and successfully recruiting psychiatric patients for clinical studies, means that there is a significant need to report which recruitment avenues were the most and least effective, to inform future clinical studies to be realistic in scope, timelines, resource assignment, and reduce the chances of research waste [18]. Here, we systematically evaluate the learnings garnered from the multimodal recruitment strategy employed by the DOCUMENT study – a comprehensive investigation of cognitive deficits in major depressive Disorder (MDD), schizophrenia (SZ) and healthy volunteers (HV). We aim to provide data-driven insights into recruitment and methodological considerations needed to successfully recruit an age-matched and ethnically representative participant sample, We collate learnings from this study into a framework designed to inform recruitment strategies for future studies.

## Methods

### Study design and setting

The DOCUMENT study (‘*Measuring cognitive deficit using cognitive tasks*’) was a single-site, two-stage, mixed-methods prospective clinical research study conducted at King’s College London. The study was designed in collaboration with, and funded by, Boehringer Ingelheim and co-sponsored by King’s College London and the South London and Maudsley (SLaM) NHS Foundation Trust. Recruitment and data collection ran over a 30-month period from June 2022 to December 2024. Ethics approval was received from the London-Camberwell St Giles Research Ethics Committee (REC reference: 21/LO/1234; IRAS ID: 304617). Full scientific results will be published once data analysis is complete.

There were two main parts to the study: Part 1 aimed to recruit 50 healthy volunteers (HV), 50 people with schizophrenia (SZ), and 75 with major depressive disorder (MDD) to undertake remote (at-home) tablet-based cognitive and (optional) speech assessments over 3 days; A subset of 25 participants in each group continued into Part 2, completing in-person clinical and cognitive tests, followed by a 14-day remote longitudinal assessment of cognitive and sleep measures. Inclusion in the Part 2 subset was offered to all participants who completed Part 1, until the Part 2 recruitment targets were fulfilled.

### Study participants and eligibility

Eligible participants were required to be adults aged 18–55 years old, fluent in English, with no intellectual disability or neurodevelopmental disorder, no concurrent participation in another study, no current serious or unstable clinically important systemic illnesses, and the capacity to provide informed consent. For Part 2, cannabis use in the prior 12 h was not permitted and was confirmed by a urine screening; for Part 1, participants were asked to abstain from all recreational drug use.

HV were required to have no current or historic psychiatric diagnoses, confirmed by a study clinician administered MINI psychiatric interview [39], no significant physical or neurological conditions, no psychotropic medication use, no diagnosed sleep disorders aside from insomnia, and no first-degree relatives with a psychotic or bipolar disorder.

MDD participants were required to have a diagnosis of depression and be experiencing a current depressive episode, verified by the study clinician using the MINI. They could not have any history of psychotic depression, primary psychotic disorders or bipolar disorders. Antidepressant medication or psychological treatments had to be stable and started > 4 weeks before enrolment. Comorbid psychiatric or neurological disorders were excluded, except for generalised anxiety disorder (GAD).

SZ participants were required to have a DSM-5 diagnosis of schizophrenia, or an unspecified schizophrenia spectrum or psychosis disorder and fit a research diagnosis of schizophrenia, which was confirmed by a study clinician and by the MINI psychiatric interview (MINI) [39]. For Part 2 eligibility, participants had to have Positive and Negative Syndrome Scale (PANSS) and Brief Negative Symptom Scale (BNSS) scores consistent with schizophrenia [19, 21]. Comorbid psychiatric or neurological disorders, aside from anxiety disorders, were excluded. SZ participants had to be free from severe symptom exacerbation requiring inpatient hospitalisation and have a diagnosis and illness duration of ≤ 10 years. Antipsychotic medication use had to be stable for the 4 weeks before enrolment in the study, with a second antipsychotic only permitted when prescribed for sleep or anxiety. Clozapine use was allowed following an amendment made to the study protocol in May 2023 (see below).

### Recruitment avenues and timeline

Participants were recruited through a multimodal recruitment strategy, incorporating clinical, institutional, online, and community outreach pathways. Recruitment avenues included NHS Clinical Services – Community Mental Health Teams (CMHTs) and early intervention services, electronic health records (SLaM Clinical Record Interactive Search (CRIS) and Consent for Contact (C4C), the NIHR BioResource (namely the Genetic Links to Anxiety and Depression (GLAD) study [8], an online recruitment platform (Call for Participants (CFP), community flyers, and word of mouth. From January 2023, the study also started using social media avenues (Meta (Facebook and Instagram) and Reddit), the NHS SLaM Take Part in Research website (slam.nhs.uk/take-part-in-research), internal King’s College London (KCL) research circulars, and previous clinically aligned research studies (PsiDer [36] and COGENT), General Practitioner (GP) Participant Identification Centres (PICs) were also used from October 2023 to send NHS text messages to potential MDD and SZ participants, inviting them to take part in the study.

**Fig. 1:**
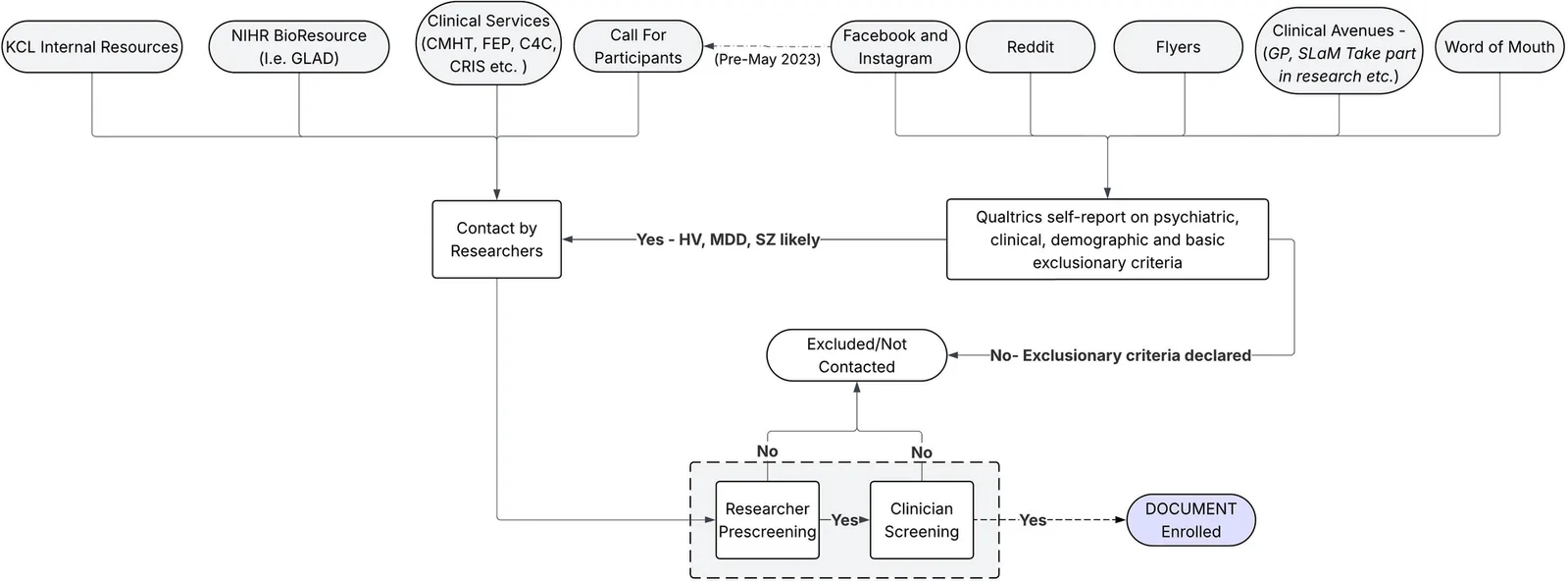
DOCUMENT Study recruitment paths. Prior to May 2023 the initial contact was made by the research team directly. From May 2023, an intermediate Qualtrics based response step was introduced to further triage for eligible individuals

Initially, participants contacted the research team directly with their interest in participating. An intermediate step was added in May 2023 in which prospective participants were directed to a secure Qualtrics web-hosted declaration of interest and basic self-report screening form using the MINI, which allowed the research team to prioritise time on screening the participants who would have otherwise only declared exclusionary criteria during a pre-screening call (Fig. 1).

### Pre-screening and eligibility confirmation

**Fig. 2:**
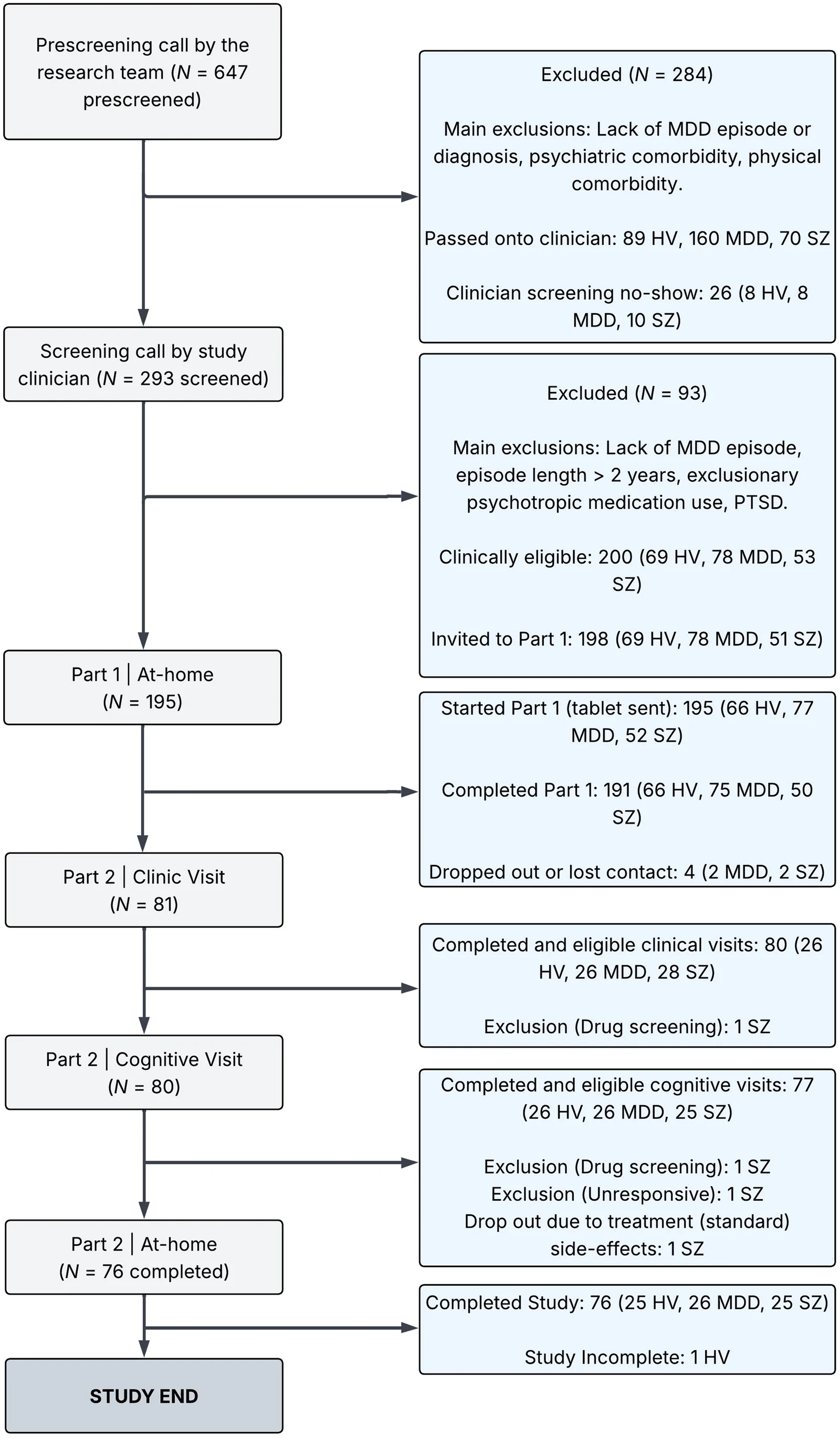
Participant and group study flow and attrition across both Parts 1 & 2 of the DOCUMENT Study. Inclusion in Part 2 of the study was offered to all participants who completed Part 1 on a ‘first-come, first served’ basis until the recruitment targets for the Part 2 subset were fulfilled

Individuals interested in participating in the DOCUMENT study underwent two calls with the study team. The first was a ~ 15-minute pre-screening call to collect demographic information and assess basic inclusion and exclusion criteria, such as self-reported psychiatric and medical history. Individuals who were considered likely to be eligible were then scheduled for a clinical screening call (~ 1 h). The clinician screening involved the administration of the MINI psychiatric interview to all screened individuals [39], alongside a review of the pre-screening answers to confirm eligibility specific to each diagnostic group. If deemed eligible by a study clinician after the screening call, participants were then invited to enrol on the study and take part in Part 1. The study participants’ progression through pre-screening, clinical screening, Part 1 and Part 2 is summarised in Fig. 2.

### Adaptive protocol amendments

Two key amendments to the study protocol were implemented following HRA/NHS REC approval in January 2023 and May 2023. Both amendments were data-driven clinical decisions to improve recruitment feasibility, reduce participant burden, and ensure the representativeness of the target diagnostic populations.

The January 2023 amendment increased the financial compensation for SZ participants from £40 to £80 in Part 1, and from £220 to £280 in Part 2, to better reflect the substantially greater clinical and time burden placed on this population, including longer screening calls and additional clinical visits. This amendment also included adding the collection of detailed ethnicity information and the highest level of education, to allow for better evaluations of sample representativeness. Accordingly, this data was not available for all enrolled participants.

The May 2023 amendment focused on broadening the eligibility criteria for SZ participants to improve recruitment feasibility and better reflect a more general population and a couple of real-world conditions. It extended the duration of diagnosis for SZ from ≤ 5 to ≤ 10 years, permitted Clozapine use, as well as antipsychotic polypharmacy for sleep or anxiety management. In addition, the requirement for a formal clinical diagnosis of SZ was relaxed to permit unspecified psychotic disorders and schizophrenia spectrum disorders if a research diagnosis of SZ was confirmed by a study clinician using the MINI [39]. For all groups, the required abstinence from cannabis before the Part 2 clinical visits was reduced from 24 to 12 h; Participants were still encouraged to abstain during the periods of remote assessment.

The study duration was initially planned as 6–12 months (aligned to REC and sponsor timelines). However, the recruitment window was extended multiple times due to a lower-than-anticipated participant accrual rate, particularly for the SZ group prior to the amendment changes.

### Study procedures and compensation

**Fig. 3:**
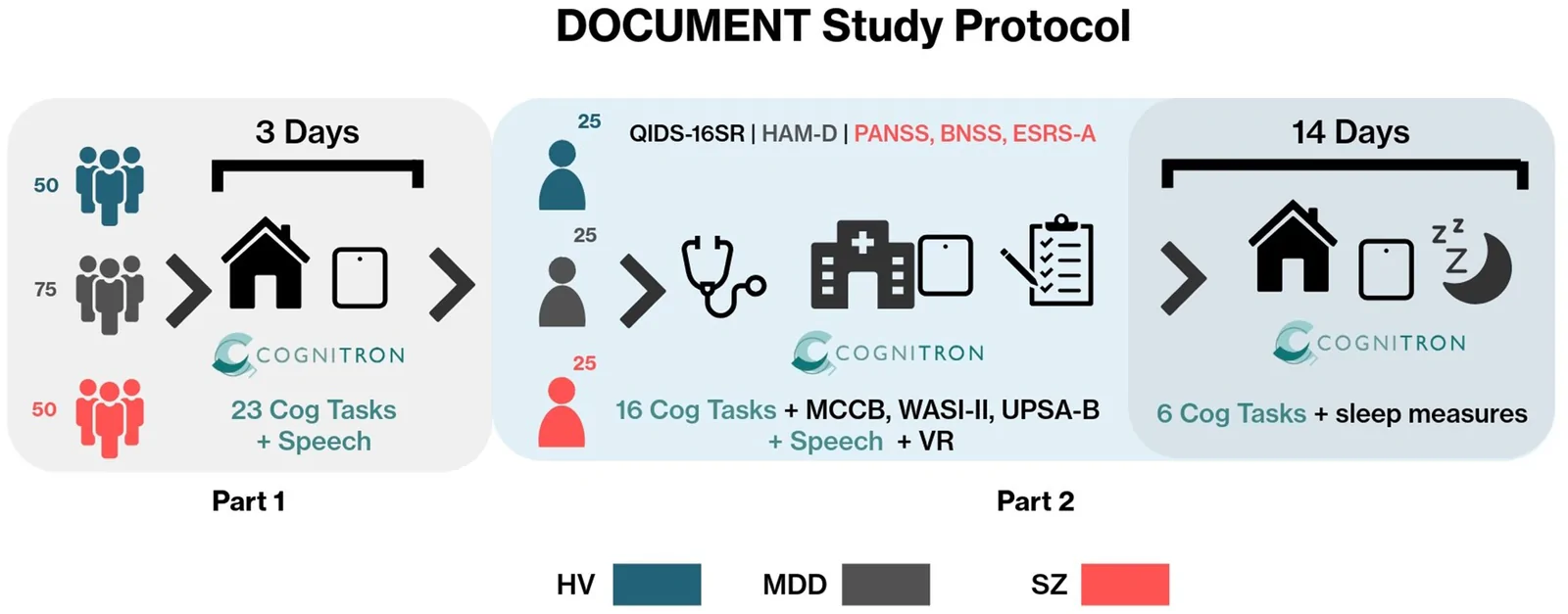
Outline of the DOCUMENT Study protocol for Part 1 and Part 2. HV – Healthy volunteers, MDD – Major Depressive Disorder participants, SZ – Schizophrenia participants. ‘Cog’ here refers to ‘Cognitive’, MCCB - MATRICS Consensus Cognitive Battery, WASI-II - Wechsler Abbreviated Scale of Intelligence (second edition), UPSA-B - University of California San Diego Performance-Based Skills Assessment (Brief)

Following enrolment into the study, participants were sent a Samsung Tablet to complete cognitive batteries via the Cognitron™ app, over two days, and optional speech sampling assessments via the Speech Vitals™ app (Linus Health), over three days (Study Part 1). Each cognitive battery took approximately 45–60 min, and the speech sampling took 10–15. Before being able to start any tasks, the participants gave informed consent via the Cognitron™ app. For successful completion, HV and MDD participants were compensated £40 and SZ participants £80. (Fig. 3).

A target of seventy-five participants (twenty-five from each group) was set for Part 2 completion. As such, eighty-one participants were invited to complete Part 2 of the study, starting the second stage within two weeks of completing Part 1. Inclusion into the Part 2 subsample required participants to have completed Part 1 within the fortnight before their first site visit, and operated primarily on a first-come, first-served basis until the group recruitment targets were fulfilled. Part 2 involved, first, an in-person clinical assessment at the NIHR King’s Clinical Research Facility within King’s College Hospital, to obtain written informed consent, and collect lifestyle factors and symptom scales by a study clinician. All diagnostic groups completed the self-report 16-item Quick Inventory of Depressive Symptomatology (QIDS-16-SR) [37]. Disorder-specific clinician-administered assessments were also undertaken: The MDD group alone underwent the clinician-administered Hamilton Depression Rating Scale (HAM-D [14], while the SZ group underwent the administration of the Positive and Negative Syndrome Scale (PANSS [19], Extrapyramidal Symptom Rating Scale-Abbreviated (ESRS-A [4] and the Brief Negative Symptom Scale (BNSS [21]. Then, a cognitive assessment visit to undergo both online (Cognitron™) and gold-standard paper-based neuropsychological cognitive batteries, optional speech sampling (Speech Vitals™), and a virtual reality (VR) based functional task. For HV and MDD participants, this typically took place on the same day, and for SZ, these visits were separate days due to longer clinical visits; With the clinical and cognitive clinic visit days within seven days of each other, typically within the same working week. At both visits, a breath alcohol concentration test and a 10-panel urine drug test were administered to rule out exclusionary criteria.

Then, at-home, over the next 14 days, the Part 2 subset of participants completed a shorter remote cognitive battery on 10 days of their choice, as well as continuous sleep measurements from a wrist-worn actigraphy watch, as well as lifestyle and sleep self-report questionnaires on the days they opted to complete the cognitive assessments [17, 33]. For Part 2 completion, the HV and MDD participants received a bank transfer of £220, and the SZ compensation was £280. The higher compensation for SZ participants across Parts 1 & 2 reflected the increased clinical and time burden (longer screening times and additional study visits) experienced by this group and was approved in the January 2023 protocol amendment by the NHS/HSC REC. Full details of the study protocol, including cognitive and clinical assessments, are in Supplement 1.

The proportion of participants completing Part 2 of the DOCUMENT study following completing Part 1 was projected to be 50% for HV and SZ (both 25/50) and 33.33% for MDD (25/75). The observed Part 1 to Part 2 progression rates were 37.88% for HV, 50% SZ, and 34.67% for MDD. To ensure the Part 2 recruitment targets were met, an additional 16 HV participants were recruited during Part 1.

### Data handling and preparation

The recruitment data were collated from across various study sources, fully anonymised and then analysed in a Jupyter Notebook (.ipynb) in Visual Studio Code (1.89.1) using Python (v3.11.5), and some descriptive statistics were carried out in Jamovi (The jamovi project, [40]). Meta-specific recruitment metrics were obtained from Meta’s Ads Manager and then pooled with the study data. Recruitment estimated costs were pooled across study invoices, emails, and records.

## Results

### Participant demographics and group recruitment rates

Over 30 months, 194 participants were recruited and enrolled in the DOCUMENT study. (Note that whilst Fig. 2 states 195 participants were sent the tablets, one participant never started data collection and was removed from the enrolled metrics).

The demographics and eligibility fractions ([enrolled participants / pre-screened participants] x 100) of the enrolled participants are outlined in Table 1. Ethnicity data were collected for 133 participants (68.56%) and education level for 131 (67.53%) following the January 2023 amendment. The three diagnostic groups were closely matched for mean age: 31.90–33.40, with the greatest distribution in the HV group (± 9.7) and the smallest in the SZ group (± 6.6). Both the HV and MDD groups had more participants with female biological sex (*F* = 72.7% HV, 61% MDD), whereas the SZ was more male-dominated (*M* = 70.6%). In the Part 2 subpopulation, the percentage of biologically female individuals became 69% in both HV and MDD groups, with an increase in the SZ group to 34%.

The median UK education level in both the HV and MDD groups was an undergraduate degree (6; IQR = 1), in the SZ group, the median was A-Level secondary education (3, IQR = 3). This difference in education was more pronounced in the Part 2 subsample, with increased median education levels of 6.5 for HV (*Bachelor’s to Master’s*) and 7 for MDD (*Master’s*), and no subsample change with 3 for SZ (*A-Level*).

**Table 1:** DOCUMENT Study enrolled participant demographics and contribution proportions of each recruitment avenue to the study and diagnostic group sample

	HV	MDD	SZ	Total
	*N* = 66	*N* = 77	*N* = 51	*N* = 194
**Age** *(Years)*				
Age Range	18–54	18–55	19–50	18–55
Mean Age (± SD)	32.06 (9.70)	33.40 (8.35)	31.90 (6.63)	32.55 (8.43)
**Sex** *(Proportion of diagnostic group sample %)*				(*Proportion of study sample %)*
Male	18 (27.27%)	30 (38.96%)	36 (70.59%)	84 (43.30%)
Female	48 (72.73%)	47 (61.04%)	15 (29.41%)	110 (56.70%)
**Median UK Education Level** *(IQR)*	6 (1)	6 (1)	3 (3)	6 (4)
**Mean Years Since Diagnosis** (± SD)	-	9.08 (7.68)	4.38 (2.87)	-
**Recruitment Avenue**,** N =** *(% of diagnostic group sample)*				(*Proportion of study sample %)*
Call For Participants (CFP)	12 (18.18%)	1 (1.30%)	-	13 (6.70%)
Clinical Services	-	-	37 (72.55%)	37 (19.07%)
Electronic Health Records	-	-	11 (21.57%)	11 (5.67%)
GP Services	1 (1.51%)	5 (6.49%)	1 (1.96%)	7 (3.61%)
KCL Internal Studies	2 (3.03%)	3 (3.90%)	-	5 (2.58%)
NIHR BioResource (e.g. GLAD)	5 (7.58%)	41 (53.25%)	-	46 (23.71%)
Meta Advertising	43 (65.15%)	25 (32.47%)	-	68 (35.05%)
SLAM NHS Take Part in Research	-	2 (2.60%)	2 (3.92%)	4 (2.06%)
Word Of Mouth	3 (4.55%)	-	-	3 (1.55%)
Pre-Screened	116	401	81	647 (49 of unknown group)
Eligible to enrol	69	78	53	200
**Eligibility Fraction**	59.48%	19.45%	65.43%	30.91%
**Study Section Attrition** *(N = Started*,* % Completion rate; Diagnostic group)*				**(***N* = Started, % Completion rate; Study sample)
**Part 1**: At-Home (Remote)	66 (100%)	77 (97.40%)	51 (98%)	194 (98.45%)
**Part 2**: (Clinic and Remote)	26 (96.15%)	26 (100%)	29 (86.21%)	81 (93.83%)

SD – Standard deviation, IQR – Interquartile range, GLAD – Genetic Links in Anxiety and Depression study, SLaM – South London and Maudsley NHS Trust, NIHR – National Institute for Health and Care Research

Out of the 194 enrolled participants to start Part 1 data collection, 191 completed this stage (Fig. 2), resulting in high retention rates of 100% for the HV group, 97.40% for MDD, and 98.04% for SZ.

A subset of 81 participants started Part 2 data collection, undergoing at least one study visit, with 76 (25 HV, 26 MDD, 25 SZ) completing both the clinical and cognitive study visits alongside the longitudinal 14-day assessment. The Part 2 retention rate was also high, with 96.15% for the HV group, 100% for MDD, and 86.21% for SZ. Full intergroup and intragroup demographic comparisons are outlined in Supplement 2, with no within-group differences observed between the Part 1 and Part 2 participant samples, and between groups the pattern of demographic differences remained consistent.

**Fig. 4:**
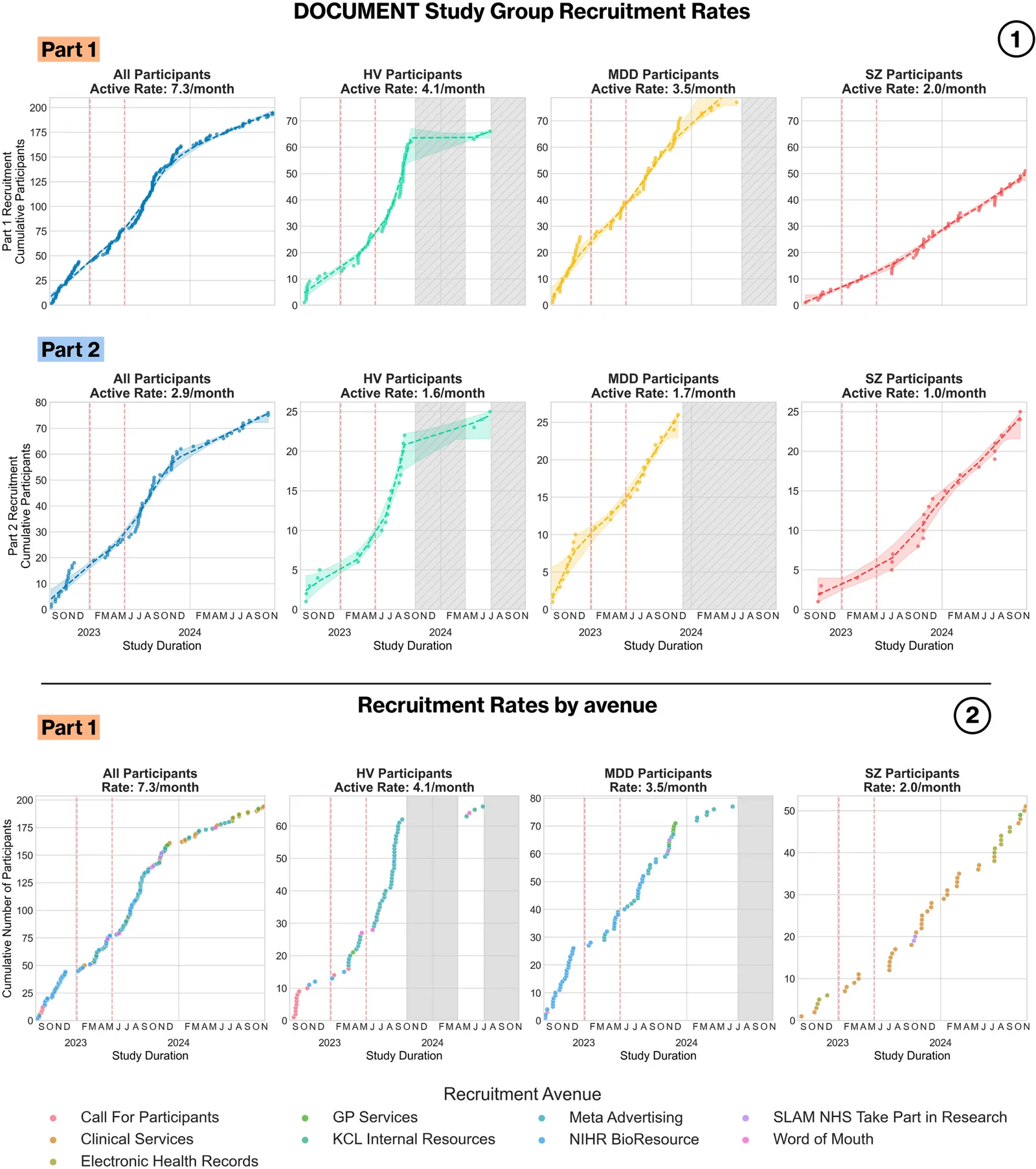
Cumulative rates of participants recruitment per group using locally estimated scatterplot smoothed (LOESS) plots for Part 1 (first line), Part 2 (second line), and by recruitment avenue (third line). The rates of recruitment have been adjusted for any pauses in recruitment at each stage per group (represented by the shaded grey areas). January and May 2023 protocol amendments are represented by vertical dashed red lines

The rate of recruitment varied between groups and between parts of the study. The first participant was enrolled in August 2022 and the last in October 2024. HV recruitment ran for 16.1 months until June 2024, with a pause between October 2023 and April 2024 to allow for targeted counterbalancing to match the demographics of the patient groups. MDD recruitment ran continuously over 21.5 months until June 2024, with recruitment for Part 2 halted in December 2023, and July 2024 for Part (1) SZ recruitment ran over 26.4 months until October 2024 for both Part 1 & (2) The Part 1 (*remote study*) recruitment rate per month across all groups was 7.3 participants, with HV at the highest rate of 4.1, then MDD at 3.5, followed by SZ having the lowest rate of 2, see Fig. 4.

The Part 2 (*remote and in-person longitudinal study*) recruitment rate was much slower across groups at 2.9 participants per month. In Part 2, MDD recruitment (1.7) was marginally higher than the HV group (1.6), with SZ still being the lowest (1).

The data-driven decision taken to amend the inclusion criteria for the SZ participants is reflected in the recruitment rates. Before the January 2023 amendment, the rate of SZ participants enrolled was 1.4 per month (*N* = 6 recruited). Between January and May 2023, the recruitment declined to 1.2 as recruitment sources were exhausted. After May 2023, the SZ rate of recruitment increased to 2.3, attributed to the cumulative criteria changes, particularly the relaxation from needing a formal clinical SZ diagnosis to allowing a research diagnosis instead.

### Demographic composition and census alignment

The ethnic makeup of the subset of the study sample for which there was ethnicity data (*N* = 133; 68.6%), closely mirrored the London ethnicity distribution outlined in the 2021 UK Census (Office for National Statistics, [31]); Table 2.

**Table 2:** Ethnicity comparison of study sample to the 2021 UK Census data for (i) London, and (ii) nationally. The demographic breakdown is based on a sample of *N* = 133 (68.6%) for which ethnicity data were available

Frequencies of Ethnicity			2021 UK Census	
Ethnicity	Counts	% of Total	London %	National %
White	67	50.37%	53.8%	81.7%
Asian	24	18.05%	20.7%	9.3%
Mixed	12	9.02%	5.7%	2.9%
Black	26	19.55%	13.5%	4.0%
Hispanic	2	1.5%	--(‘*Other*’: 4.4%)	--(‘*Other*’: 1.6%)
Arab	2	1.5%	1.9%	0.7%

A post-hoc sensitivity analysis was carried out to assess whether the composition of the retrospectively collected (*N* = 28) and post- May 2023 amendment (*N* = 105) data significantly differed. A Pearson’s *χ*^2^ test of independence showed no significant difference between the samples: *χ*^2^(5, *N* = 133) = 10.21, *p* = .07. Cramér’s *V* = 0.277 also indicated a small-to-moderate but non-significant shift in ethnicity distribution.

Within the diagnostic groups, notable differences in ethnicity composition were apparent (Table 3). The HV group in both Part 1 and 2 were predominantly white and asian, whereas in the MDD group, the sample was largely white. The SZ sample, in contrast, included higher proportions of black participants than the other groups. Across all groups, participants with mixed, hispanic, and arab ethnicities were less represented in the study population, compared with the other ethnic groups.

**Table 3:** Ethnicity representation across participant groups (HV, MDD, SZ) for the study sample and sub-sample. The proportion of missing data was calculated for each diagnostic group and study phase. The ethnicity counts and proportions were calculated from where participant data were available

*= with available data				Part 2 Sub-sample		
Ethnicity	HV	MDD	SZ	HV	MDD	SZ
Counts*=	44	48	41	19	14	23
White	20 (45.5%)	36 (75.0%)	11 (26.8%)	8 (42.1%)	11 (78.6%)	5 (21.7%)
Black	2 (4.5%)	2 (4.2%)	22 (53.7%)	2 (10.5%)	1 (7.1%)	14 (60.9%)
Asian	19 (43.2%)	3 (6.3%)	2 (4.9%)	9 (47.4%)	1 (7.1%)	0 (0%)
Mixed	1 (2.3%)	6 (12.5%)	5 (12.2%)	0 (0%)	1 (7.1%)	3 (13.0%)
Hispanic	1 (2.3%)	1 (2.1%)	0 (0%)	0 (0%)	0 (0%)	0 (0%)
Arab	1 (2.3%)	0 (0%)	1 (2.4%)	0 (0%)	0 (0%)	1 (4.3%)
*Missing data*	22 (33.3%)	29 (37.7%)	10 (19.6%)	7 (26.9%)	12 (33.3%)	6 (20.7%)

### Recruitment avenue comparison

**Fig. 5:**
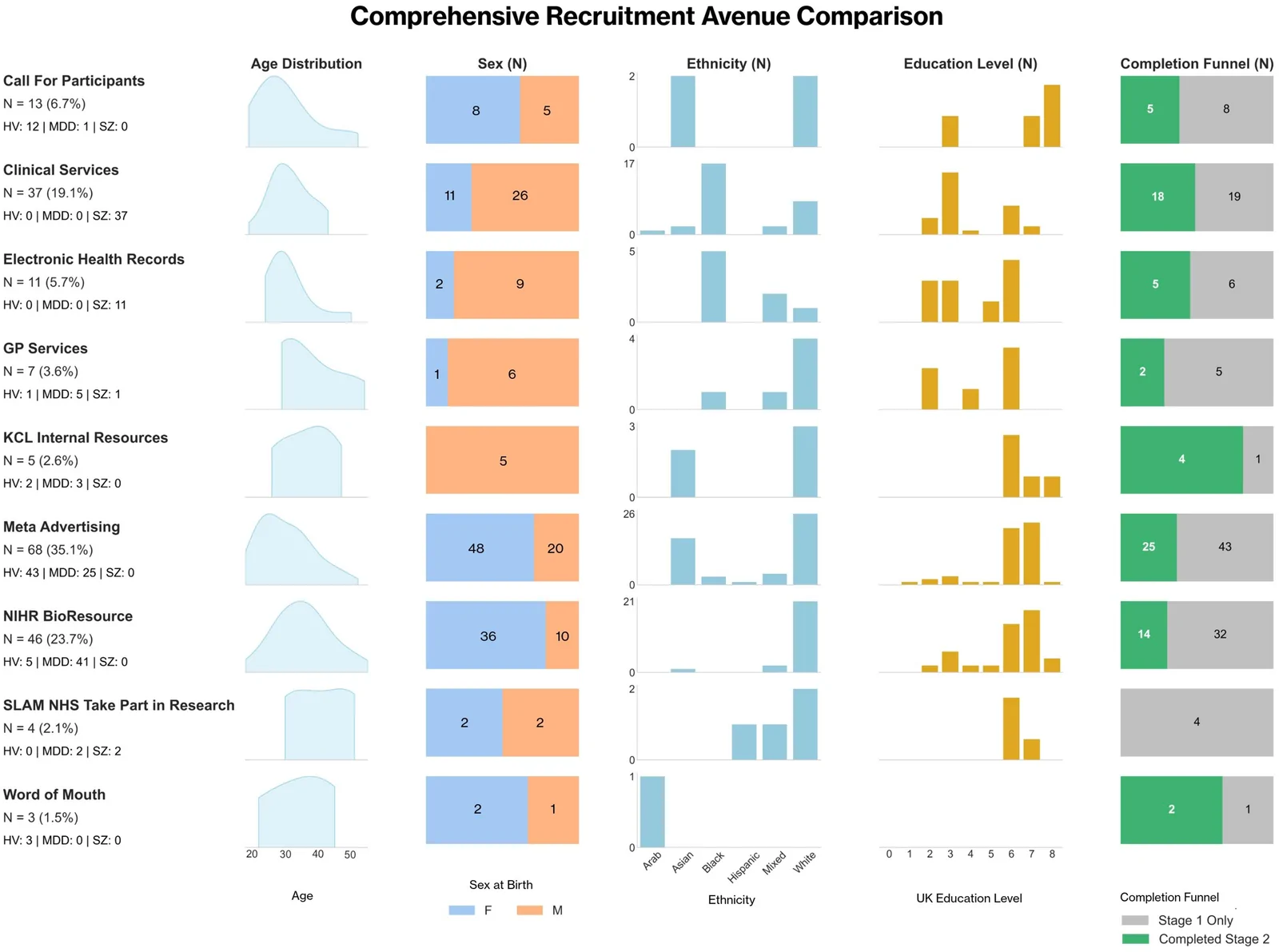
A comprehensive recruitment avenue comparison of diagnostic group yield, age, sex, ethnicity, education, and study Part1-Part2 completion

Recruitment avenues differed in their contribution to diagnostic groups, eligibility fractions, and demographic composition. Each avenue is summarised below for total yield, eligibility fraction, efficiency, and demographic characteristics, with full details in Tables 1 and 4, and visualised in Fig. 5.

The majority of the HV participants were recruited through Meta advertising (65.15%), CFP (18.18%) and the NIHR BioResource (7.58%). For MDD participants, the dominant sources were the NIHR BioResource (53.25%), Meta advertising (32.47%) and GP services (6.49%). The SZ recruitment came predominantly from clinical services (72.55%), EHRs (21.57%), and the SLaM Take Part in Research site (3.92%).

Full granular recruitment avenue comparisons are provided in Supplement 3, with the most pertinent avenue-specific findings provided below:

### Meta advertising

The largest contributor to the overall study sample was Meta advertising (35.1%), yielding 43 HV and 25 MDD participants, which had the second-highest recruitment rate across the avenues (3.3 participants per month) and a moderate eligibility fraction (47.7%). However, this avenue failed to yield any SZ participants, despite targeted efforts at a cost of £538.25. The participants from this avenue were predominantly female (70.6%) and younger adults (30.3 ± 8.2).

### Clinical pathways (CMHTs and EHRs)

Clinical pathways, namely CMHTs and EHRs, yielded the highest proportion of SZ participants across all recruitment avenues (94.1%) with a high eligibility fraction (73.3–77.1%). This route carried a high degree of unquantified labour costs – such as time spent searching through patient records and liaising closely with the clinical care teams. This avenue additionally proved unsuccessful for MDD recruitment (via the Clinical Record Interactive Search) due to co-morbidity exclusions. Participants through these avenues were predominantly male (70.3–81.8%), with both clinical services (31.6 ± 6.2) and EHRs (31.6 ± 7.4) having similar mean ages, and greater ethnic diversity than the online avenues.

### NIHR BioResource

The NIHR BioResource (i.e. GLAD) recruited the highest proportion of MDD participants. It had the highest recruitment rate across avenues (3.6 participants per month), but the lowest eligibility fraction (14.5%), necessitating the adoption of intermediate pre-screening for basic exclusionary criteria through Qualtrics. This avenue also produced the strongest demographic skew, with the majority of patients from this avenue being female (78.3%, white (87.5%) and marginally older than some of the other avenues (35.5 ± 8.7). Whilst no direct financial cost was incurred by the study directly, the labour cost for this resource was provided by the NIHR.

### GP-PIC sites

GP-PIC across 15 sites yielded a small proportion of the study population (3.6%), with 5 MDD, 1 SZ and 1 HV, at a rate of 1.5 per recruitment attempt – discounting the untargeted enrolled HV participant. This route had a low eligibility fraction (22.6%), despite a total spend of £2,700 through the NIHR Research Delivery Network (RDN). The resultant sample was older (37.4 ± 10.1), white (66.7%), and predominantly male (85.7%); However, this is not reflective of the actual potential due to targeted sex counterbalancing.

### Call for participants (CFP)

CFP yielded a small proportion of the study sample (6.7%) at a negligible total cost (£40), with a relatively high eligibility fraction (68.4%; 12 HV, 1 MDD), but a low recruitment rate of 1.04 participants per month. Participants through this avenue were generally younger adults (30.5 ± 9.6) and female (62%). However, 69% of the CFP participants had missing education or ethnicity data, due to the timing of this avenue falling predominantly before the amendment that collected these additional demographics.

### Internal university resources

KCL Internal university resources (circular emails and previous studies), yielded 2.6% of the study sample (2 HC, 3 MDD). The recruitment rate was moderate at 1.16 participants per month, with an eligibility fraction of 38.46%. Participants via this avenue were exclusively male, and predominantly white (60%) or asian (40%).

### SLaM take part in research

SLaM Take Part in Research recruited 2.1% of the study population (2 MDD, 2 SZ) at no direct cost. There was a low recruitment rate (0.25 participants per month) but a high eligibility fraction of 80%. Participants via this avenue were on average older (40.8 ± 10.3) than the other avenues, and an even male-to-female split.

### Word of mouth

Word of mouth yielded 3 HV participants (1.6% of the study population), with the highest eligibility fraction (100%), but a low recruitment rate (0.17 participants per month). Notably, this avenue wasn’t actively pursued, and the participants recruited were aged 34.3 ± 11.6, and the majority were female (66.7%).

### Other approaches

Community-focused flyering and poster efforts were also attempted with no success. Reddit advertisements were also tried at a sunk cost of £94.68 with no yield, due to respondents fulfilling exclusionary comorbid or physical disorder criteria and being filtered out at the Qualtrics triaging stage. Due to multiple avenues feeding concurrently into the triaging step, the exact number of Reddit advertisement respondents could not be accurately determined.

**Table 4:** Recruitment Avenue recruitment rate and cost-per-enrolled participant, with total spend and eligibility fraction

	HV *N* = 66	MDD *N* = 77	SZ *N* = 51	Total *N* = 194	
	Rate (Cost per enrolled participant)			Rate (Total spent)	Eligibility Fraction
Call for Participants (CFP)	0.96 (£3.08)	0.08 (£3.08)	-	1.04 (£40)	68.42%
Clinical Services*	-	-	1.41*	1.41	77.08%
Electronic Health Records*	-	-	0.49*	0.49	73.33%
GP Services	-	2 (£427.82)	1 (£133.09)	1.5 (£2,700)	22.58%
Institution (KCL) Internal Studies	1.96	0.66	-	1.16	38.46%
NIHR BioResource (e.g. GLAD)*	0.50*	3.18*	-	3.56	14.51%
Meta Advertising	3.66 (£4.10)	1.59 (£25.47)	- (£538.25)	3.29 (£1,351.29)	47.68%
SLAM NHS Take Part in Research	-	0.18	0.13	0.25	80%
Word of Mouth	0.17	-	-	0.11	100%
Reddit Advertising	-	-	-	- (£94.68)	-
*Group Rates and Cost (Rate per month*,* Cost per enrolled participant)*					
**Part 1**: At-Home (Remote)	4.1	3.5	2	7.3	
**Part 2**: (Clinic and Remote)	1.6	1.7	1	2.9	
Cost per enrolled	£3.35	£42.19	£14.04*	£21.58	
Total Group Spend	£221.18	£3,248.56	£716.23	£4,185.97	

* = Significant unquantified labour costs involved

### Sex-disparity of recruitment costs

Across the recruitment avenues, there was also a higher average cost per enrolled (CPE) male (£41.87) than female (£5.22) participants. The cost associated with recruiting participants with either male or female assigned sex at birth was calculated by the total spend on that recruitment avenue divided by the number of enrolled participants assigned to the targeted sex at birth; No participants with assigned sex at birth as ‘Other’ were enrolled in the study. Full comparisons of the sex disparity in recruitment costs across each diagnostic group are outlined in Supplement 4.

### Benefits of intermediate triage

The intermediate Qualtrics basic pre-screening step saved the research team approximately 84.25 h of manual screening calls of 337 individuals who would have been considered ineligible, saving an estimated £1,802.64 in labour costs. Across recruitment avenues, Qualtrics received 1,079 total responses, of which 893 (82.76%) were unique respondents. Notably, 65 individuals (7.27%) submitted multiple completed responses, highlighting the necessity for robust duplicate response checking.

The step also offered key insights into the diagnostic profiles of ineligible participants. These were: 29 individuals with anxiety, without a MDD or Psychosis diagnosis (8.61%), 95 with post-traumatic stress disorder (28.19%), 81 with an eating disorder (24.04%), 63 with an autism spectrum diagnosis (63%), 92 with attention deficit hyperactivity disorder (27.3%), 50 with dyslexia (14.84%), and 64 with another psychiatric comorbid disorder (18.99%).

Across all recruitment avenues, 647 individuals were pre-screened by the research team, of whom 319 (49.30%) were then invited to be screened by a study clinician (Fig. 2). Eligibility rates differed substantially by group, with higher eligibility for SZ (65.43%) and HV (59.48%) than MDD (19.45%). The main exclusions for HV individuals were undisclosed psychiatric or otherwise exclusionary medical history and ineligible lifestyle factors (e.g. nightshifts). For MDD individuals, the main exclusionary reasons were failure to meet the criteria for a current ongoing episode, exclusionary comorbid conditions, and excessive episode duration. The main SZ screening exclusions were exclusionary substance use and failure to reach the criteria for a research SZ diagnosis. Detailed exclusionary characteristics are reported in Supplement 5.

### Clinical and cognitive characteristics

No significant associations between recruitment avenue and participants’ MDD or SZ medication type or class were identified. Full comparisons of time since first diagnosis and medication use by diagnostic group and recruitment avenue are in Supplement 6.

Due to the focus of the study on cognitive performance, exploratory analyses examined whether changes in recruitment strategy over the study duration introduced unintended sampling bias in task accuracy, whilst controlling for demographic and clinical covariates; The full analysis is presented in Supplement 6. No significant within-group differences in cognitive performance were identified. When pooled across groups, a slight decline in total population cognitive performance was observed over time, but this was likely reflective of the later focus on recruitment of people with SZ rather than recruitment avenue effects.

### Recruitment efficiency

**Fig. 6:**
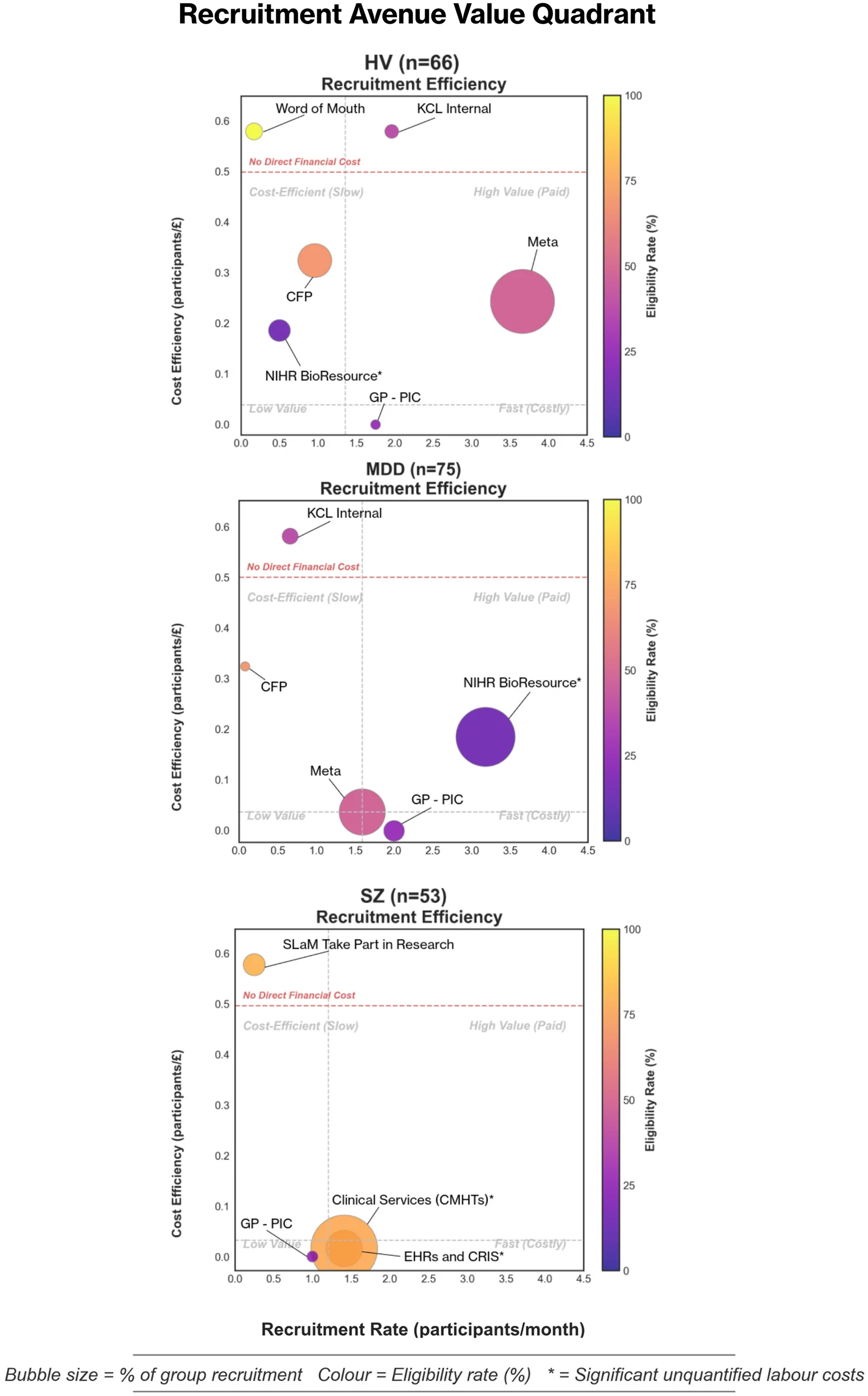
A value quadrant of recruitment avenues, time-based and financial efficiency, eligibility rates, and proportional contributions, based on the research insights from the case-control DOCUMENT study. Recruitment avenues with no direct financial cost are plotted above the dashed red line for visual distinction

The collective avenue-specific findings are synthesised in Fig. 6, integrating time and financial efficiency, eligibility fraction, and the proportional diagnostic group sample contribution as a value quadrant for recruiting HV, MDD and SZ participants. Figure 6 displays the time and financial efficacy trade-off for each avenue (each axis), the proportion of participants recruited by said avenues for the diagnostic group (bubble size), and the eligibility quotient of participants from each avenue (bubble colour gradient). To better reflect the labour costs associated with the Clinical Service and EHR avenues, an estimated hourly rate of £21.45 has been calculated at a rate of 2 h per enrolled participant. For the NIHR BioResource, this inherent labour cost has been estimated at 15 min per enrolled participant.

## Discussion

Here, we took a data-driven, adaptive, and multimodal approach to support representative recruitment of individuals with MDD and SZ, as well as healthy volunteers. Whilst timeline extensions and protocol amendments were notably necessary, particularly for the SZ group, the study populations were age-matched, achieved demographic representativeness aligned with the London 2021 UK Census (Office for National Statistics, [31]), and achieved high retention across both study phases (85.7–100%). These findings serve as a transparent account of how recruitment challenges can be identified early, monitored and mitigated in ways that may support sample representativeness and reduce research waste.

Recruitment efficiency across avenues differed substantially across the DOCUMENT study, and demonstrated multidimensional trade-offs between speed, financial and labour efficiency, and eligibility fractions. Meta advertising showed high recruitment rates and low per-participant costs, but a low eligibility fraction. In contrast, traditional clinical and electronic health record (EHR) pathways were much slower and had a substantial labour cost but also showed a higher eligibility fraction. Divergence in the effectiveness of recruitment avenues also differed by diagnostic group. Online approaches were highly effective for both HV and MDD participants, aligned with prior findings of this avenue as successful in recruiting affective disorder populations [13, 25]. In contrast, SZ recruitment was almost entirely reliant on traditional clinical pathways (94%), consistent with prior reports of clinical pathway dependency in this population [9, 35], and potentially reflecting the clinical severity, functional impairment, and digital access barriers experienced by this population, limiting access to engagement with digital online recruitment efforts [15]. Despite targeted attempts at online recruitment for this population, no eligible participants were enrolled through this avenue. The lack of clinically severe patients - SZ – recruited through online avenues corroborates the existing literature that online avenues are less efficacious due to limiting factors such as digital access issues and stigma [15], which compound with the existing constraint of fluctuating ability to participate, compromised insight, and remote diagnostic verification [35]. However, it was not possible to empirically determine whether this lack of efficacy despite efforts for SZ recruitment through Meta advertising was due to an algorithmic failure in identifying qualifying individuals, or if there was a systemic lack of engagement with the Meta adverts by this population, either due to possible mistrust or access issues. With no singularly efficacious avenue across all diagnostic groups, these results therefore support the utilisation of hybrid recruitment models to combine online recruitment for low-cost, high-yield recruitment of less clinically severe conditions, with the high-labour cost, slower traditional clinical pathways for severe and complex clinical populations. As notably visualised in Fig. 6, no single recruitment avenue simultaneously occupies the high-value quadrant of the figure across the diagnostic groups, visually reinforcing the necessity of utilising a hybrid-avenue recruitment strategy.

The overall DOCUMENT study population is notably well-aligned to the London demographics from the 2021 UK census. The skew in the DOCUMENT sample towards a population with an assigned sex at birth of female in the MDD group (61%) and male in the SZ group (71%) is consistent with London and UK-wide epidemiological patterns, with females being 1.39 times more likely to have a depressive disorder [1], and males being 1.04 to 2.3 times more likely to have a diagnosis of SZ [20, 30]. The multimodal recruitment strategy adopted here may have helped mitigate the sampling biases and demographic skews inherent in over-reliance on single pathways. Thus, the NIHR BioResource, from which over half of the MDD participants were recruited, is disproportionally white and female, contrasting with the higher incidence of common mental disorders – including depression – in non-white populations [43], but in line with the underrepresentation of minorities in registry-based recruitment [15]. Similarly, Meta advertising, whilst recruiting a more ethnically diverse population, also maintained a female skew, in line with known online recruitment biases [25]. In contrast, clinical avenues for SZ recruitment yielded a predominantly black clinical population reflective of the increased incidence of schizophrenia-spectrum and first-episode psychosis (FEP) diagnoses in Black and Minority Ethnic (BME) groups, particularly in London [20, 30]. Nevertheless, whilst aligned with higher incidence rates, the proportion of BME participants in this sample is slightly higher than general population estimates [5, 30], in line with the overrepresentation of BME participants in psychosis and FEP research noted by Michaels et al., [28]. Collectively, these findings emphasise the critical importance of adopting hybrid and multimodal recruitment strategies, alongside continuous monitoring, to actively counterbalance sampling biases.

Monitoring of the eligibility fraction, exclusionary reasons, and recruitment rates of different avenues and diagnostic groups allowed for the informed relaxation of SZ eligibility criteria over the course of DOCUMENT study, which almost doubled the rate of recruitment within this group. Furthermore, awareness of the main exclusionary reasons across groups at the pre-screening stage informed the adoption of the intermediate triaging of respondents via Qualtrics, saving an estimated 84.25 h of time that was able to be deployed elsewhere in the study. These findings illustrate how taking an adaptive and data-driven approach to recruitment can enhance feasibility and representativeness and ensure that resources are most effectively deployed.

Our results further highlight that realistic research planning for clinical research should consider both direct financial costs and the potential for substantial labour demands. Consideration and awareness of these trade-offs between labour, cost, speed and representativeness, as illustrated in Fig. 6, are important considerations for realistic and feasible clinical study planning. Furthermore, aiming for representativeness can come with an uncosted premium. We found that in the pursuit of aiming to recruit a more balanced sex split across groups resulted was an 8-fold increase in the cost per enrolled participant in males (£41.87) compared to females (£5.22), and in MDD participants, this was an almost 24-fold increase (£100.35 male to £4.19 female). Sex-based disparities in recruitment cost were also observed across both Call for Participants and Meta advertising. Therefore, our results make apparent that there is an – often uncosted – ‘representativeness tax’ of both financial and labour costs that are required for obtaining balanced and representative study samples.

Even with successful initial recruitment, high participant attrition rates across clinical studies can still cause a study to fail [2]. Notably, the high retention rate across both parts of the DOCUMENT study is also contrary to the high attrition rates frequently reported in interventional trials in psychiatry [16]. This may be reflect a combination of factors, such as maintaining personalised and frequent contact with the participants and offering flexible scheduling for study visits, consistent with prior literature [7]. Notably, over the course of the DOCUMENT study, a dedicated member of the research team regularly engaged with clinical teams and caregivers to build trust and communication with the research team to facilitate SZ recruitment. This approach may partly reflect the higher proportion of SZ clinical service recruitment compared to EHR screening alone, as well as the high retention rate observed, even in this complex clinical population.

Synthesising these collective findings from 30 months of recruitment on the DOCUMENT study, we propose a 10-point framework (Table 5) to apply for representative and efficient clinical research, grounded in empirical observations. Whilst recruitment avenue specific yields and demographics may differ by locality, clinical population and healthcare system, the framework principles may still be applicable across clinical and research contexts, however the extent of their transferability are likely dependent upon local resources, clinical infrastructure, and the characteristics of the population under study.

### Clinical recruitment framework

Where the recommendations made in the clinical recruitment framework (Table 5) are based directly on the empirical findings reported in the study, these are designated as such. Additionally, where any recommendations are based upon informed inferences, anecdotal research experience, and the wider literature, this is also indicated to support appropriate interpretation.

**Table 5:** A data-driven framework for representative and adaptive recruitment in psychiatric research studies, based upon insights from the DOCUMENT study

Recruitment framework	Implications for recruitment	Recommendation basis
i. Diagnosis-specific recruitment strategy	*Recruitment avenues should match the engagement by the target population*,* rather than a one-size-fits-all approach.*	Recruitment yield differed considerably across both diagnostic groups and avenue; Fig. 6 highlights the lack of a one-size-fits all approach.
ii. Multimodal recruitment for representative populations	*Multimodal recruitment can help to sidestep inherent single-source recruitment biases and increases the diversity and representativeness of study populations.*	The close alignment of the DOCUMENT study population to the 2021 UK Census for London despite the recognised single-avenue sampling biases. However, the causal contribution of multimodality is inferred rather than empirically tested.
iii. Real-time demographic counterbalancing and monitoring	*Counter recruitment and sample bias by continuous monitoring of demographic drift and dynamically adjust recruitment strategy in response.*	The study used frequent and considered demographic monitoring and counterbalancing, such as adapting MDD recruitment to focus on underrepresented Males or the HV recruitment pause for more targeted sampling from HV recruitment pause October 2023 to April 2024, but this was operational rather than tested as a formal intervention.
iv. Data driven adaptability of the study protocol	*Monitor study data to identify barriers to study recruitment and retention that may be adapted without compromising the integrity of the research.*	The data-driven January and May 2023 study amendments made to the inclusion/exclusion criteria and compensation tangibly increased the rate of recruitment; from 1.2 SZ per month to 2.3 post-amendment.
v. Implementing digital pre-screening infrastructure	*Intermediate digital self-report triaging systems (such as Qualtrics)*,* between recruitment avenue and the research team*,* asking likely exclusionary criteria pre-screening questions can reduce ineligible pre-screening rates.*	The Qualtrics triaging step saved an estimated 84.25 h and £1,802.64 in labour costs by filtering out respondents who had study exclusionary characteristics.
vi. Clinical pathways focus for severe psychiatric disorders	*Clinically severe psychiatric disorders*,* such as SZ*,* require more clinically focused pathways for recruitment*,* and recruitment and retention is substantially improved by building trust and rapport with the patients*,* caregivers and service providers. Digital-first approaches are likely insufficient for these means.*	The majority of SZ recruitment came overwhelmingly through clinical routes (CMHTs and EHRs), with digital routes – aside from the NHS SLaM take Part in Research website - failing to recruit eligible SZ participants. The impact of building rapport with the subjects, and its effect on recruitment and retention, is principally anecdotal.
vii. Transparent reporting of recruitment	*Clear reporting of recruitment sources*,* eligibility rates*,* exclusions*,* time or financial efficiency*,* and demographics by avenue increase reproducibility*,* as well as the generalisability of research findings.*	Whilst not directly empirically tested in the DOCUMENT study, the transparent reporting of the differences in recruitment efficacy, financial and labour costs, and demographic characteristics, clearly identify the avenue specific sampling biases and variable diagnostic-group efficacy. This suggestion aligns with the established literature outlining the necessity for transparent reporting [18, 42]
viii. Strategic recruitment avenue allocation	*Use recruitment avenues intentionally*,* serving an operational purpose depending on the study needs*,* such as filling a representativeness gap in a study population. E.g. Digital-based methods are often fast and broad but only suited for certain populations (here: HV and MDD).*	Based on observed avenue-specific trade-offs. Such as Meta for yield and faster recruitment for HV-MDD, clinical for targeting SZ, and GP-PIC for targeted counterbalancing (such as for Male MDD).
ix. Realistic disorder-based recruitment forecasting	*Recruitment timelines and study resource allocation should reflect the diagnosis-specific feasibility*,* e.g. severe psychiatric disorders (such as SZ) may have much slower and unpredictable rates than HV and MDD.*	The recruitment rates clearly differed by group and study phase, such as being especially slower for the SZ sample recruitment.
x. Design for realistic populations, not perfect samples	*Study designs for realistic clinical profiles – such as high comorbidity of conditions – improves recruitment feasibility and representativeness. Overly strict criteria may exclude much of the real-world clinical population.*	An empirically informed inference, supported by the relaxing of inclusion/exclusion criteria and the subsequent improvement in SZ recruitment yield and recruitment rate after the Jan/May 2023 amendments. Also somewhat identified by the reported main Qualtrics and pre-screening exclusionary reasons, but a formal testing of the principle is inferential.

Whilst the current recruitment insights and framework are derived from a UK and London-centric context, many of the barriers to recruitment for clinical research are widely considered global experiences and not unique to psychiatric research. Whilst clinical, institutional, regulatory and epidemiological factors may vary across countries, the tension between recruitment speed, financial and labour costs, eligibility, and representativeness remains pervasive. Therefore, the underlying framework emphasising multimodal recruitment pathways, demographic monitoring, and data-driven protocol amendment is likely transferable across broader clinical research contexts.

Several limitations of the present work should be acknowledged. First, the insights of the study are drawn from the context of a single UK site, and in the recruitment of participants with MDD/SZ or as HVs. Therefore, the generalisation of these findings – particularly the proposed recruitment framework - is most appropriately applied to similar research or clinical populations, and the extent to which they apply to other inclusion/exclusion criteria, different clinical and non-clinical populations and multicentre studies is yet to be determined. The DOCUMENT study was an observational clinical study conducted in a locality that is uniquely situated for psychosis and psychiatric recruitment and incidence [20], the exact efficacy of recruitment avenues in different settings may be tied to local resources and clinical incidence rates that are different from the present study. Furthermore, whilst the barriers to recruitment largely overlap, it should also be acknowledged that randomised control trials may encounter additional constraints that come with interventional studies [29], such as more rigid inclusion criteria. Likewise, this study is not an exhaustive list of possible recruitment avenues, as strategies such as Google advertisements, transport advertisements and radio could have been utilised [23, 44]. In addition, other studies have shown success with online approaches to SZ recruitment that were not replicated by the present study [10]. Additionally, due to concurrent online recruitment avenues feeding into the Qualtrics triaging step, avenue-specific bot-generated or inauthentic responses could not be formally established, particularly considered to be a risk of non-identity linked platforms such as Reddit. However, despite these limitations, it is expected that the framework put forth in this study for adaptive and representative clinical research recruitment should still be widely applicable and beneficial.

Successful recruitment is foundational to the representativeness, validity, and translational impact of clinical research. With transparent and granular reporting of recruitment challenges, adaptations, and their outcomes, the DOCUMENT study aims to provide a comprehensive evaluation of real-world psychiatric recruitment. The data-driven framework proposed herein supports the shift from a one-size-fits-all approach to recruitment and towards more efficient and hybrid research practices that aim to reduce research waste from failed recruitment and may have relevance and applicability beyond UK-based psychiatric research, however replication of these insights in other clinical and research populations and contexts is needed.

## Supplementary Information

Below is the link to the electronic supplementary material.

Supplementary Material 1

## Data Availability

Due to the presence of clinical and sensitive demographic data, the datasets generated and analysed in the present study are not publicly available. However, an anonymised version of the datasets, along with the Jupyter Notebook (Python) analysis code, may be made available upon reasonable request to the corresponding author, subject to institutional, ethical and GDPR governance requirements.

## Abbreviations

ADHD: Attention Deficit Hyperactivity Disorder
BME: Black and Minority Ethnicities
BNSS: Brief negative Symptom Scale
C4C: Consent For Contact
CFP: Call For Participants
CMHT: Community Mental Health Teams
CONSORT: The Consolidated Standards of Reporting Trials
CRIS: Clinical Record Interactive Search
DOCUMENT: Measuring cognitive deficits using cognitive tasks study
DSM-5: Diagnostic and Statistical Manual of Mental Disorders, Fifth Edition
ESRS-A: The Extrapyramidal Symptom Rating Scale
FEP: First Episode Psychosis
GAD: Generalised anxiety disorder
GLAD: Genetic Links to Anxiety and Depression
GP: General Practitioner
HAM-D: Hamilton Depression Rating Scale
HV: Healthy Volunteers
HTA: UK Health Technology Assessment
KCL: King’s College London
MDD: Major Depressive Disorder
MINI: Mini-International Neuropsychiatric Interview
MRC: Medical Research Council
NHS: National Health Service (UK)
NIHR: National Institute for Health and Care Research
PANSS: Positive and Negative Syndrome Scale
PIC: Participant Identification Centres
PTSD: Post-Traumatic Stress Disorder
QIDS-16-SR: The 16-item Quick Inventory of Depressive Symptomatology- Self Report
REC: Research Ethics Committee
SLaM: South London and Maudsley NHS Foundation Trust
SZ: Schizophrenia
