# Supplementary material for: A data-driven methodological framework for representative recruitment in psychiatric research: insights from the DOCUMENT study
Source: BMC Psychiatry. 2026 Jul 31;26:759. doi: 10.1186/s12888-026-08442-x (PMC13643963; doi:10.1186/s12888-026-08442-x)
Supplement: Supplementary file 1 [file 12888_2026_8442_MOESM1_ESM.docx]

# Supplementary

## Supplement 1 – Study Methods

***Study protocol and compensation***

As outlined in the study **Methods**, the DOCUMENT study consisted of two main parts. In part one of the study, the aim was to recruit 50 HV, 50 SZ, and 75 MDD participants for remote cognitive assessments. Enrolled participants were sent a Samsung Galaxy Tab A8 10.5” tablet, or a Samsung Tab A7 10.4” LTE tablet with a 4G SIM, to ensure device hardware standardisation and maximise inclusivity. The participants were asked to complete assessments over 3 days. On days 1 and 2, the participants completed a battery of 11 cognitive tasks each day (22 in total, a block ‘A’ and a block ‘B’) on their tablets covering a variety of domains via the Cognitron™ app, with participants randomly counterbalanced to ‘A’ or ‘B’ conditions to control for which 11 tasks were completed first (A-B, or B-A). The exact tasks and target domains for each stage can be found in **Supplementary** **Table 1**. The participants were provided instructions and asked to complete the tasks between 6-9 pm, with the assessments taking ~45 minutes each day. On days 2 and 3, the participants could also optionally complete a speech sampling assessment using the Linus Health Speech Vitals App.

Reimbursement for Part 1 completion was cash or an Amazon voucher to the value of £40 for HV and MDD, and £80 SZ (£40 pre-January 2023). In total, 66 HV, 50 SZ and 75 MDD participants completed Part 1 of DOCUMENT.

|  |  | DOCUMENT Study Stage | | |
| --- | --- | --- | --- | --- |
| Cognitron Task | **Relative Domain** | **Part 1** (*Remote*) | **Part 2a** (*Clinic*) | **Part 2b** (*Remote*) |
| **Prospective Memory** | *Memory* | x | x | x |
| **Spotter** | *Attention* | x | x | x |
| **Block Design** | *Spatial planning, Working memory, Executive Function* | x | x |  |
| **Digit Span** | *Numeric short-term memory* | x | x |  |
| **Switching Stroop** | *Cognitive control, Attention, Executive function* | x | x | x |
| **Verbal Analogies** | *Semantic (analogical) reasoning, Verbal reasoning, Problem solving* | x | x |  |
| **Forager** | *Exploit decision-making, Adaptability* | x | x | x |
| **Tic Tac No** | *Response Inhibition* | x |  |  |
| **Precision 2-Back** | *Working memory, Attention* | x | x | x |
| **Mallas Memory** | *Working memory, Executive function* | x |  |  |
| **Tower Of London** | *Spatial planning, Working memory* | x |  |  |
| **Lead Balloon** | *Motor coordination* | x | x |  |
| **Spatial Span** | *Spatial short term memory capacity, Working memory* | x |  |  |
| **Manipulations 2D** | *Visuospatial reasoning, Perceptual-Motor function, 2D mental rotations, Spatial planning, Working memory* | x | x |  |
| **Cognitron Gambling Task** | *Risk management* | x | x |  |
| **Beads** | *Decision making and probability assessment* | x | x |  |
| **Cipher** | *Symbolic association, working memory and processing speed* | x | x |  |
| **Dual 2-Back** | *Working memory, Affective control* | x |  |  |
| **Digit Goal Neglect** | *Feature discrimination, following the rules* | x | x |  |
| **Simple Reaction Time** | *Processing speed, Motor Reaction time* | x |  |  |
| **Choice Reaction Time** | *Processing speed, Motor Reaction time* | x |  |  |

**Supplementary Table 1:** Overview of Cognitron™ administered tasks by study stage and Cognitive domain.

Part 2 of the study was an in-depth, clinic-based follow-up with multimodal longitudinal assessments conducted on a subset of participants who completed Part 1, aiming for 25 subjects per group. Within two weeks of completing Part 1, participants attended, first, a clinical assessment visit at the NIHR King's Clinical Research Facility (CRF) located in King’s College Hospital – part of the South London and Maudsley NHS Foundation Trust. During the visit, participants gave written consent, then underwent a breath alcohol concentration test and a 10-panel urine drug screening test to rule out exclusionary criteria. Positive results on either resulted in exclusion or rescheduling of the visit. Participants’ weight, blood pressure, pulse and lifestyle factors (caffeine, alcohol or substance use) were recorded. The study clinician carried out brief physical and neurological examinations and reviewed any current and prior medications.

All participants completed the self-report Quick Inventory of Depressive Symptomatology (QIDS-16-SR). MDD participants additionally underwent the clinician-administered Hamilton Depression Rating Scale (HAM-D). SZ participants underwent the clinician-administered PANSS (Positive and Negative Syndrome Scale), BNSS (Brief Negative Symptom Scale), and ESRSA (Extrapyramidal Symptom Rating Scale - Abbreviated). The assessments took around 30 minutes for HV, 45 minutes for MDD, and 2 hours for SZ. Due to the increased duration for SZ, this clinical assessment visit was typically on a separate day from the cognitive visit, and the lifestyle factors were reviewed at this second visit.

The cognitive visit also took place at the CRF, with participants first undergoing a 14-task battery of Cognitron™ app-administered assessments on the tablets, taking approximately 45 minutes. The display order of the tasks depended on the participants’ randomised ‘A’ or ‘B’ counterbalancing from Part 1. Trained members of the research team then administered paper-based neuropsychological batteries, the MATRICS Consensus Cognitive Battery (MCCB, ~60 minutes), Wechsler Abbreviated Scale of Intelligence - Second Edition (WASI-II, ~45 minutes), and the UCSD Performance-Based Skills Assessment - Brief Version (UPSA-B, ~15 minutes), in this order and taking breaks between batteries as needed by the participants. Speech sampling data was also collected using the Samsung tablets if participants had opted in during Part 1. Participants also completed a virtual reality (VR) assessment of simulated real-world cognitive demands in a virtual supermarket environment, using an HTC Vive™ headset with handheld controllers. They were guided through a training session for control and environment familiarity before the assessment.

Over the following 14 days, participants’ sleep and activity patterns were assessed using the wrist-worn ActiGraph CentrePoint® Insight Watch (CPW01). On 10 days of the participants’ choice, they were also asked to complete a shorter Cognitron™ battery of 5 tasks between 6-9 pm, and to complete a series of sleep (COSMOS, Epworth Sleep Scale- ESS) and lifestyle questions when they woke up on each day. As mentioned, participants received a £220 bank transfer as reimbursement for their participation. Following the January 2023 amendment, the SZ group received £280.

## Supplement 2 – Between-within demographic comparisons of Part 1 study sample and Part 2 subsample

Between and within-group demographic differences were investigated using independent samples t-tests (Welch) for age – analysis of variance (ANOVAs) in the intergroup compartisons, Mann-Whitney U for UK education level and small group sizes, Fisher’s exact for sex at birth, and chi-squared for ethnicity.

**Within-disorder sample demographic comparison**

Notably, no significant differences in the demographics of the Part 1 study sample and Part 2 sub-sample were observed within any of the participant groups; see **Supplementary Tables 2 to 6** below.

*Healthy Volunteers*

| **Demographic** | **HV (Part 1)** | **HV (Part 2)** | **P1 – P2 comparison** |
| --- | --- | --- | --- |
| ***N* =** | 66 | 25 |  |
| **Age, Mean (±SD)** | 32.1 (± 9.7) | 32.7 (±9.4) | *t(*89) = -0.30, *p* = 0.768 |
| **Sex assigned at birth , N (%)** |  |  | Fisher's *p* = 0.795 |
| Female | 48 (72.7%) | 17 (68.0%) |  |
| Male | 18 (27.3%) | 8 (32.0%) |  |
| **Ethnicity, N (%)** |  |  | χ²(5) = 2.34, *p* = 0.801 |
| White | 20 (30.3%) | 7 (28.0%) |  |
| Black | 2 (3.0%) | 2 (8.0%) |  |
| Asian | 19 (28.8%) | 9 (36.0%) |  |
| Hispanic | 1 (1.5%) | - |  |
| Arab | 1 (1.5%) | - |  |
| Mixed | 1 (1.5%) | - |  |
| Missing | 22 (33.3%) | 7 (28.0%) |  |
| **UK Education Level, Median (range)** | 6 (1-8) | 7 (1-8) | U = 389, *p* = 0.648 |

**Supplementary Table 2:** Healthy volunteer demographic characteristics between the Part 1 study sample and Part 2 sub-population.

*Major Depressive Disorder*

| **Demographic** | **MDD (Part 1)** | **MDD (Part 2)** | **P1 – P2 comparison** |
| --- | --- | --- | --- |
| ***N* =** | 77 | 26 |  |
| **Age, Mean (±SD)** | 33.4 (±8.4) | 33.0 (±7.5) | *t*(101) = 0.21, *p* = 0.836 |
| **Sex assigned at birth , N (%)** |  |  | Fisher's *p* = 0.491 |
| Female | 47 (61.0%) | 18 (69.2%) |  |
| Male | 30 (39.0%) | 8 (30.8%) |  |
| **Ethnicity, N (%)** |  |  | χ²(4) = 0.80, *p* = 0.939 |
| White | 36 (46.8%) | 11 (42.3%) |  |
| Black | 2 (2.6%) | 1 (3.8%) |  |
| Asian | 3 (3.9%) | 1 (3.8%) |  |
| Hispanic | 1 (1.3%) | - |  |
| Arab | - | - |  |
| Mixed | 6 (7.8%) | 1 (3.8%) |  |
| Missing | 29 (37.7%) | 12 (46.2%) |  |
| **UK Education Level, Median (range)** | 6 (2-8) | 7 (3-8) | U = 254, *p* = 0.341 |

**Supplementary Table 3:** Major Depressive Disorder demographic characteristics between the Part 1 study sample and Part 2 sub-population.

*Schizophrenia*

| **Demographic** | **SZ (Part 1)** | **SZ (Part 2)** | **P1 – P2 comparison** |
| --- | --- | --- | --- |
| ***N* =** | 51 | 25 |  |
| **Age, Mean (±SD)** | 31.9 (±6.6) | 30.7 (±5.8) | *t*(74) = 0.79, *p* = 0.431 |
| **Sex assigned at birth , N (%)** |  |  | Fisher's *p* = 0.606 |
| Female | 15 (29.4%) | 9 (36.0%) |  |
| Male | 36 (70.6%) | 16 (64.0%) |  |
| **Ethnicity, N (%)** |  |  | χ²(4) = 1.82, *p* = 0.769 |
| White | 11 (21.6%) | 4 (16.0%) |  |
| Black | 22 (43.1%) | 13 (52.0%) |  |
| Asian | 2 (3.9%) | - |  |
| Hispanic | - | - |  |
| Arab | 1 (2.0%) | 1 (4.0%) |  |
| Mixed | 5 (9.8%) | 3 (12.0%) |  |
| Missing | 10 (19.6%) | 4 (16.0%) |  |
| **UK Education Level, Median (range)** | 3 (2-7) | 3 (2-6) | U = 421, *p* = 0.732 |

**Supplementary Table 4:** Schizophrenia demographic characteristics between the Part 1 study sample and Part 2 sub-population.

These intragroup comparisons indicate a demographic continuity between the overall Part 1 study sample and the Part 2 subsamples, for age, sex, ethnicity and education.

**Between-disorder sample demographic comparison**

Across all diagnostic groups, in both Part 1 and Part 2 samples, there were no significant differences in age, indicating successful matching: Part 1 - *F*(2, 191) = 0.65, p = .521, Part 2 - *F*(2, 73) = 0.67, *p* = .514. Assigned sex at birth, however, significantly differed across the groups in both the Part 1 (*p* < .001) and Part 2 samples (*p* = .026). Likewise, ethnicity also significantly differed across all participant groups in Part 1 and Part 2 samples (*p* < .001). UK education level also significantly differed across the participant groups for Part 1 and Part 2 (p < .001).

*Part 1 Demographics*

| **Demographic** | **HV** | **MDD** | **SZ** | **Group comparison** |
| --- | --- | --- | --- | --- |
| ***N* =** | 66 | 77 | 51 |  |
| **Age, Mean (±SD)** | 32.1 (±9.7) | 33.4 (±8.4) | 31.9 (±6.6) | *F*(2,191) = 0.65, *p* = 0.521 |
| **Sex assigned at birth , N (%)** |  |  |  | χ²(2) = 22.96, *p* < .001 |
| Female | 48 (72.7%) | 47 (61.0%) | 15 (29.4%) |  |
| Male | 18 (27.3%) | 30 (39.0%) | 36 (70.6%) |  |
| **Ethnicity, N (%)** |  |  |  | χ²(10) = 74.18, *p* < .001 |
| White | 20 (30.3%) | 36 (46.8%) | 11 (21.6%) |  |
| Black | 2 (3.0%) | 2 (2.6%) | 22 (43.1%) |  |
| Asian | 19 (28.8%) | 3 (3.9%) | 2 (3.9%) |  |
| Hispanic | 1 (1.5%) | 1 (1.3%) | - |  |
| Arab | 1 (1.5%) | - | 1 (2.0%) |  |
| Mixed | 1 (1.5%) | 6 (7.8%) | 5 (9.8%) |  |
| Missing | 22 (33.3%) | 29 (37.7%) | 10 (19.6%) |  |
| **UK Education Level, Median (range)** | 6 (1-8) | 6 (2-8) | 3 (2-7) | *F*(2,128) = 19.62, *p* < .001 |

**Supplementary Table 5:** DOCUMENT Study Part 1 intergroup demographic comparison

*Part 2 Demographics*

| **Demographic** | **HV** | **MDD** | **SZ** | **Group comparison** |
| --- | --- | --- | --- | --- |
| ***N =*** | 25 | 26 | 25 |  |
| **Age, Mean (±SD)** | 32.7 (±9.4) | 33.0 (±7.5) | 30.7 (±5.8) | *F*(2,73) = 0.67, *p* = 0.514 |
| **Sex assigned at birth , N (%)** |  |  |  | χ²(2) = 7.33, *p* = 0.026 |
| Female | 17 (68.0%) | 18 (69.2%) | 9 (36.0%) |  |
| Male | 8 (32.0%) | 8 (30.8%) | 16 (64.0%) |  |
| **Ethnicity, N (%)** |  |  |  | χ²(8) = 37.22, *p* < .001 |
| White | 7 (28.0%) | 11 (42.3%) | 4 (16.0%) |  |
| Black | 2 (8.0%) | 1 (3.8%) | 13 (52.0%) |  |
| Asian | 9 (36.0%) | 1 (3.8%) | - |  |
| Hispanic | - | - | - |  |
| Arab | - | - | 1 (4.0%) |  |
| Mixed | - | 1 (3.8%) | 3 (12.0%) |  |
| Missing | 7 (28.0%) | 12 (46.2%) | 4 (16.0%) |  |
| **UK Education Level, Median (range)** | 7 (1-8) | 7 (3-8) | 3 (2-6) | *F*(2,49) = 15.12, *p* < .001 |

**Supplementary Table 6:** DOCUMENT Study Part 2 intergroup demographic comparison

Whilst intergroup differences in demographic variables were observed in both the Part 1 and Part 2 group samples, the pattern of these differences was consistent across both parts, highlighting the likelihood that the Part 2 subsample is an accurate and representative group from which to draw inferences.

## Supplement 3 – Granular Recruitment Avenue Comparison

Cost per enrolled participant here is simply:

[*Cost of recruitment avenue / Number of enrolled participants from that avenue*]

Age and education level across recruitment avenues and diagnostic groups is visualised in **Supplementary Figure 1** below.

**Call For Participants**

The CFP site was used by the DOCUMENT study between August 2022 to August 2023, costing £40 for two recruitment ads during this time. The adverts placed were aimed at recruiting all three diagnostic groups. Over this period, 19 individuals were pre-screened by the research team (13 HV, 5MDD), of which 13 (68.42%; 12 HV, 1 MDD) were deemed eligible and enrolled in the study. All 13 subsequently completed Part 1 of the study, and 5 HV started and completed Part 2. This equated to £3.08 per enrolled participant, with a recruitment rate of 1.04 participants per month.

It should be noted that 69% of the CFP participants had no education or ethnicity data.

**Meta Advertising**

Advertising on both Facebook and Instagram with Meta’s advertising platform was also used extensively for recruitment between February 2023 and October 2024, with a high total cost of £1,351.29. Over 20.7 months, 14 ads were run on 136 separate days (310 ad days total – where advertisement days overlapped). Ads were primarily static 1080 x 1080p images, as video use was stopped after poor A|B testing, and the only audience specifications were 18-55 years old within 50km of London. The ads ran on average for 22 days, costing £4.36 per day (an average of £96.52 total per ad run). At this time, the average reach of an ad was 18,411 individuals, not considering repeat viewership, and 4,399 link clicks to the Qualtrics site. The recruitment ads reached 257,759 unique individuals, with each ad shown ~1.67 times per individual.

151 individuals were pre-screened by the research team, and 72 were deemed eligible (47.68%). Of these, 68 participants were enrolled via this avenue into the study: 43 HV and 25 MDD. The recruitment rate was the highest of all avenues, with an average of 3.3 participants per month over 20.7 months. The cost per enrolled participant was £19.87. 67 participants completed Part 1 (98.53%; 43 HV, 34 MDD), 26 started Part 2 (16 HV, 10 MDD), and 25 completed both parts (96.15%; 15 HV, 10 MDD).

Of the 14 ads, 4 were jointly targeting HV-MDD, 5 MDD-SZ, 2 only SZ, and 3 only MDD participants. The total amount spent targeting each of the participant groups was £176.29 HV, £636.76 MDD, and £538.25 SZ. However, despite a concerted effort, no SZ participants were successfully recruited via this avenue. When, therefore, considering the relative HV and MDD spending alone, the cost per eligible participant became £4.10 per HV and £25.47 per MDD. Furthermore, due to counterbalancing efforts to try and identify male MDD participants, 2 of the 12 MDD adverts were targeted at this population, accounting for £242.72 of the total costs.

The Meta advertisement enrolled sample had 25% with missing ethnicity and education data.

**Clinical Avenues: EHRs and Clinical Services**

Clinical recruitment avenues, such as clinical services (e.g. CMHTs, First Episode Psychosis services) and patient electronic health records, yielded 94.12% of the 51 schizophrenia participants for the study (72.55% and 21.57% respectively).

Recruitment through these avenues ran the entire study duration, and no specific costs were associated with advertising the study through this avenue. However, a dedicated member of the DOCUMENT study team led the identification and recruitment of people with schizophrenia via this route. Building rapport and trust between the research team and the clinical care team for the participants cannot be quantified here but should be noted as a crucial labour and time cost. Notably, approaching and screening the patient caseloads from the community clinical services had a much higher rate of recruitment (1.4 per month) than just EHR (Clinical Record Interactive Search - CRIS) screenings (0.5 per month).

48 people with SZ from community clinical services were pre-screened, and 37 were deemed eligible (77.08%). EHRs had a comparable eligibility rate at 73.33%, with 15 pre-screened and 11 eligible. The higher eligibility rate in SZ screenings compared to HV and MDD is due to the research team typically having access to notes on SZ participants’ relevant medical history and liaising with their consultants and care coordinators for input on their eligibility before screening. 37 SZ from clinical services and 11 from EHRs were enrolled in the study, with 36 and 11 (97.3-100%) completing Part 1; The participant dropped out before starting Part 1, and so the 36 are counted in the enrolled demographics. From clinical services and EHRs, 22 and 6 SZ started Part 2, and 19 and 5, respectively, completed the study (83.3-86.7%).

26.31% Clinical Service and EHR-enrolled participants are missing ethnicity and education data.

**GP Services**

At 4 timepoints, 15 General Practices (GP) across South London were used as Participant Identification Centres (PICs) for participant recruitment, costing a total of £2,700, funded by the NIHR Research Delivery Network (RDN). These GP sites were located across the boroughs of Southwark, Croydon, Lambeth and Greenwich. 3,356 MDD-targeting recruitment text messages were sent by the sites in October 2023 and January 2024, with the January texts being follow-ups to the recipients of the October texts. 174 SZ-targeting text messages were sent at two periods in September 2024.

31 individuals were pre-screened by the research team, with 7 eligible (22.58%) and enrolled in the study: 5 MDD, 1 SZ, and – rather surprisingly - 1 HV. Notably, the HV participant seemed to be recruited because of the MDD batch texts by the GP sites. Compared to the other avenues, GP-PIC site recruitment per eligible participant was much more expensive than the other avenues at an average of £385.71, particularly given the lower rate of eligibility into the study. Of the 7 enrolled participants, 6 completed Part 1 (85.71%; 1 HV, 4 MDD, 1 SZ), and 2 (1 MDD, 1 SZ) started and completed Part 2 of the study (100%). The GP-PIC recruitment rate, using each ad period as a proxy measurement for months, was 1.75 eligible participants.

The GP-PIC texts, as mentioned, were used as an effort to recruit more male MDD participants (as these were harder to come by), and so £2,566.91 was spent with this aim. This worked out at £427.82 per MDD (and HV) participant enrolled. The SZ GP texts were less specific and worked out at £133.09 per SZ participant enrolled in comparison, though this only yielded a single subject. Each GP-PIC site carried out a search using limited criteria of their databases to match the study inclusion and clinical exclusion criteria.

16.67% of the GP-enrolled participants have no ethnicity or education data.

**NIHR BioResource**

Recruitment using GLAD ran from August 2022 to September 2023, with a rate of 3.56 enrolled participants per month. The avenue was targeted for MDD participants, but due to the nature of the GLAD study, some HV participants were also recruited via this avenue.

317 individuals were pre-screened from GLAD, of which just 46 (14.51%) were eligible: 5 HV, 41 MDD. These participants then all completed Part 1, and 14 (1 HV, 13 MDD) started and completed Part 2 of the study.

Ethnicity and education data are not available for 47.83% of the participants enrolled by the NIHR BioResource avenue.

**Internal University Resources**

King’s College London university internal research circular emails and research studies, such as the KCL-BI COGENT study (cognitive performance in healthy volunteers), KCL Psilocybin in Depression Resistant to Standard Treatments (PsiDeR) study, and the KCL internal recruitment circular email, were also utilised and have been combined into a single insight umbrella group for cohesion and comparison to the other recruitment methods.

From August 2023 to May 2024, 13 individuals were pre-screened, and 5 (2 HV, 3 MDD) were enrolled in the study (1.16 per month; 38.46% eligibility). All participants completed Part 1, and 4 (2 HV, 2 MDD) started and completed Part 2.

**SLaM Take Part in Research**

National Health Service (NHS) based online patient self-recruitment platforms, such as the South London and Maudsley NHS Foundation Trust Take Part in Research site (slam.nhs.uk/take-part-in-research), were another source of study advertisement that was used towards the end of the DOCUMENT study. Recruitment through SLaM Take Part in research ran from September 2023 to November 2024, with a recruitment rate per month of 0.25. The avenue was targeted at all three diagnostic groups.

**Supplementary Figure 1:** Age and Education demographic differences across DOCUMENT study recruitment avenues. The size of the data points is relative to the size of the group-specific sample from each avenue. The dashed line indicates the mean across all groups for age and UK education level, respectively.

## Supplement 4 – Disparity in sex-based recruitment costs by diagnostic group

Across all three diagnostic groups, males were also more expensive per enrolled participant, costing £5.79 per HV (*F* = £2.25), £100.35 per MDD (*F* = £4.19), and £133.09 per SZ; However, the only effective direct SZ spend was for a single participant enrolled through a GP site who was male.

The lower cost of female-based assigned sex at birth recruitment was also identified in the per avenue breakdowns. Call for Participants yielded an overall CPE of £4 per male and £2.50 per female. Comparatively, Meta advertising had a CPE of £39.85 per male and £11.55 per female. In the HV group, Meta CPE costs were £8.81 per male and £2.67 per female. Whereas in the MDD group, this was even more pronounced, with Meta CPE costs of £43.97 per male and £13.13 per female.

## Supplement 5 – Exclusionary Reasons

In addition to recruitment avenues, another important consideration for successful recruitment is understanding the main exclusionary factors from the target clinical populations.

In total, the research team pre-screened 647 (116 HV, 401 MDD, 81 SZ, 49 Unknown) individuals, and passed on 319 (89 HV, 160 MDD, 70 SZ) to the study clinician as likely to be eligible, making the eligibility quotients at the researcher stage 49.30%; 76.72% HV, 39.90% MDD, 86.42% SZ.

293 clinician screenings using the MINI psychiatric interview were conducted by the study clinician (81 HV, 152 MDD, 60 SZ), of which 200 were considered potentially eligible to participate in the study (69 HV, 78 MDD, 53 SZ). This then put the confirmatory clinical pre-screening eligibility quotient at 68.26% (85.19% HV, 51.32% MDD, and 88.33% SZ).

At the research team’s pre-screening stage, the most common reasons for HV ineligibility were: History of depression (N = 4, 12.12%), regularly working nightshifts (N = 4, 12.12%), anxiety (no MDD) or panic disorder (N = 2, 6.06%), and long covid (N = 2, 6.06%). In comparison, at the clinician screening stage, the main exclusionary reasons were also: History of depression (N = 6, 50%), and sole anxiety (N = 2, 16.67%).

In MDD participant pre-screenings by the research team, the main exclusionary factors were: Not being in a depressive episode (N = 49, 21.4%), episode length of > 2 years (N = 13, 5.68%), chronic pain or fatigue condition (N = 11, 4.8%), receiving hormone replacement therapy (N = 10, 4.37%), not having a formal MDD diagnosis (N = 9, 3.98%), and a sleep disorder other than insomnia (N = 10, 4.37%). The main exclusionary criteria at the clinician screening level, in contrast, were: Not in a depressive episode (N = 20, 27.78%), episode length of > 2 years (N = 17, 23.61%), and ongoing or past (complex-) post-traumatic stress disorder diagnosis (N = 5, 6.94%).

In comparison, the main exclusionary factor from the SZ researcher pre-screenings was: Likely-substance abuse disorder (N = 2, 25%). The exclusions at the clinician level were qualitatively different, with the main reasons being: Not having a SZ diagnosis supported by the MINI (N = 2, 22.22%) and likely substance abuse disorder (N = 2, 22.22%).

## Supplement 6 – Medication and Clinical characteristics by group and avenue

Further exploratory analysis also identified the mean number of years since a diagnosis of major depressive disorder as 9.08 (± 7.68; *N* = 75). In the schizophrenia sample, this was 4.38 (± 6.63; *N* = 51); As observed in **Supplementary Figure 2**.

**Supplementary Figure 2**: Schizophrenia and major depressive disorder participant duration since diagnosis of disorder, split by group (Panel 1) and recruitment avenue (Panel 2).

The May 2023 amendment to allow a retrospective research diagnosis of SZ and extending the diagnosis duration from a maximum of 5 years to 10 years did not cause much fluctuation to the mean diagnosis length. Before the amendment, the average was 4.36 years (± 2.62) and post-amendment this became 4.39 (± 2.97).

To understand whether the clinical composition of the participants changed with the recruitment avenue changes, correlational analyses were run for the MDD and SZ groups, against the study duration There was a moderate-to-weak negative change in the MDD diagnosis length for the Part 1 sample (*r* (75) = -0.32, *p* = .006), but not the Part 2 (*r* (22) = -0.33, *p*. = .113). This indicated that as the study went on, the MDD participants’ time since diagnosis became lower – likely resultant from the Meta participants having a lower mean diagnosis duration than the GLAD participants (**Supplementary Figure 2**). No significant changes in the SZ participants’ time since diagnosis were observed over the course of the study (*p* = .528-.801 for Parts 1 & 2), even with the amendment.

Medication class and drug type for both the MDD and SZ participants in both study phases, and split by avenue, can be found in **Supplementary Figure** **3**.

To further assess whether recruitment avenues yielded clinically different participant samples, a series of chi-square independence tests was conducted to identify whether medication class and type varied significantly by research avenue.

**Supplementary Figure 3:** Medication class and type by recruitment avenue in the DOCUMENT study main MDD and SZ patient samples

No significant associations were observed between medication class and status in MDD participants and recruitment avenue: χ²(18, *N* = 76) = 23.42, *p* = .175. Though due to some avenues having < 5 cases, the results here should be interpreted cautiously. Likewise, in the SZ group, no significant associations between recruitment avenue and medication class characteristics were observed either: χ²(4, *N* = 50) = 1.34, *p* = .854.

## Supplement 7 – Cognitive changes by group and avenue

As the DOCUMENT Study’s main aim was to identify cognitive deficits across the participant groups, additional exploratory analyses were conducted to understand whether the dynamic changes to the recruitment avenues and strategy as the study progressed impacted the overall cognitive composition of the diagnostic groups in question between the start and end of the study.

An accuracy performance composite was derived across the Cognitron™ cognitive tasks using exploratory factor analysis by extracting the first unrotated factor. The procedure aggregated the task-level variations into a single measure of general accuracy. This accuracy (ACC) composite was then regressed against the study duration, controlling for age, sex, ethnicity, diagnosis duration, medication (class and polypharmacy in MDD and SZ), education (pre-university 0-3, undergraduate 4-6, postgraduate 7-8) and task counterbalancing order, across each group and combined (**Supplementary Figure 4**).

**Supplementary Figure 4:** Cognitive task accuracy (ACC) across the duration of the study by (1) diagnostic group, and (2) recruitment avenue. The red dotted lines denote the January and May amendments to the study. The grey sections identify periods where recruitment for each patient group was halted.

No significant changes in cognitive task accuracy (ACC) were observed across the main study sample for any single group (*p* = .110 - .563). Collectively across all three groups, there was a significant decrease in cognitive accuracy over the duration of the study (*b* = -.001, *t*(59) = -2.06, *p* =.043), indicative of the bulk of the psychosis group primarily being recruited towards the latter end of the study: *F*(7, 59) = 8.30, *p* = < .001, adj-R^2^ = .44; Thereby an expected methodological result, and not likely to be reflective of quantitative bias in the recruitment methods themselves.

Likewise, in the Part 2 sub-sample, the task accuracy composite (ACC) did not differ significantly over the study duration within participants in the HV (*p* = .375) or SZ (*p* = .233) groups in the regression analyses – both in the controlled and uncontrolled models for covariates. Notably, the MDD regression model indicated a significant change in ACC within the group over the course of the study (*b* = -0.005, *t*(1) = -41.60, *p* = .015). However, upon further inspection, due to the number of covariates (*k* = 15) being higher than the subsequent final sample when accounting for MDD participants with all available data (*n* = 12), the model was overspecified, and are reported here for transparency, but should otherwise be considered statistically meaningless: F(10, 1) = 1817.45, *p* = .018, adj-R^2^ = .99. The MDD uncontrolled regression model did not find there to be a significant change in the ACC across time (*p* = .944).

No significant differences in the ACC composite for each group were observed (*p* = .169 - .664) between the recruitment avenues using two separate Analyses of Covariance (ANCOVA) – one for the overall study sample, and one for the Part 2 subsample – using the same control variables as the regression.
